# Importance of Windows of Exposure to Maternal High-Fat Diet and Feto-Placental Effects: Discrimination Between Pre-conception and Gestational Periods in a Rabbit Model

**DOI:** 10.3389/fphys.2021.784268

**Published:** 2021-11-25

**Authors:** Delphine Rousseau-Ralliard, Marie-Christine Aubrière, Nathalie Daniel, Michèle Dahirel, Gwendoline Morin, Audrey Prézelin, Jérémy Bertrand, Catherine Rey, Pascale Chavatte-Palmer, Anne Couturier-Tarrade

**Affiliations:** ^1^Université Paris-Saclay, UVSQ, INRAE, BREED, Jouy-en-Josas, France; ^2^Ecole Nationale Vétérinaire d’Alfort, BREED, Maisons-Alfort, France; ^3^Université Paris-Saclay, INRAE, UE SAAJ, Jouy-en-Josas, France; ^4^ViroScan3D SAS, Trevoux, France

**Keywords:** fetal programming, placenta, liver, fatty acids, gene expression, rabbit, high fat diet

## Abstract

**Context and Aim:** Lipid overnutrition in female rabbits, from prepuberty, leads to impaired metabolism (dyslipidemia and increased adiposity) and follicular atresia, and, when continued during gestation, affects offspring phenotype with intrauterine growth retardation (IUGR) and leads to placental and lipid metabolism abnormalities. Growth retardation is already observed in embryo stage, indicating a possible implication of periconceptional exposure. The objective of this study was to discriminate the effects of preconception and gestational exposures on feto-placental development.

**Materials and Methods:** Rabbit 1-day zygotes were collected from female donors under control (CD) or high-fat-high-cholesterol (HD) diet and surgically transferred to the left and right uterus, respectively, of each H (*n* = 6) or C (*n* = 7) synchronized recipients. Close to term, four combinations, CC (*n* = 10), CH (*n* = 13), HC (*n* = 13), and HH (*n* = 6), of feto-placental units were collected, for biometry analyses. Fatty acid (FA) profiles were determined in placental labyrinth, decidua, fetal plasma, and fetal liver by gas chromatography and explored further by principal component analysis (PCA). Candidate gene expression was also analyzed by RT-qPCR in the placenta and fetal liver. Data were analyzed by Kruskal–Wallis followed by Dunn’s pairwise comparison test. Combinations of different data sets were combined and explored by multifactorial analysis (MFA).

**Results:** Compared to controls, HH fetuses were hypotrophic with reduced placental efficiency and altered organogenesis, CH presented heavier placenta but less efficient, whereas HC presented a normal biometry. However, the MFA resulted in a good separation of the four groups, discriminating the effects of each period of exposure. HD during gestation led to reduced gene expression (nutrient transport and metabolism) and big changes in FA profiles in both tissues with increased membrane linoleic acid, lipid storage, and polyunsaturated-to-saturated FA ratios. Pre-conception exposure had a major effect on fetal biometry and organogenesis in HH, with specific changes in FA profiles (increased MUFAs and decreased LCPUFAs).

**Conclusion:** Embryo origin left traces in end-gestation feto-placental unit; however, maternal diet during gestation played a major role, either negative (HD) or positive (control). Thus, an H embryo developed favorably when transferred to a C recipient (HC) with normal biometry at term, despite disturbed and altered FA profiles.

## Introduction

The incidence of obesity and overweight has reached epidemic levels in the United States, Europe, and other developed countries worldwide (Ng et al., 2014). Caused by food transition and changes in dietary behavior (over-nutrition of industrialized food), this metabolic status is associated with a major risk of developing serious diet-related non-communicable diseases, such as type-2 diabetes, cardiovascular disease, hypertension, and stroke, that are often associated with non-alcoholic fatty liver disease and reduce the overall quality of life and overall lifespan. Recent estimates show that worldwide, more than 21% of women of childbearing age will be obese by 2025 (Poston et al., 2016). Excess body weight reduces fertility and increases obstetric complications, such as miscarriage, gestational diabetes, hypertension, pre-eclampsia, and difficult delivery (Langley-Evans, 2009). In addition, children born to obese or overweight mothers have increased adiposity and are at higher risk of metabolic diseases later (Mitanchez and Chavatte-Palmer, 2018).

Most studies exploring the effects of maternal over-nutrition during pregnancy have focused on the second and/or third trimester of pregnancy, by which time key processes such as organogenesis are completed. The specific effect of pre-conception over-nutrition has been less studied (Fleming et al., 2004; Kwong et al., 2006), although there is clear evidence from animal and a limited number of human studies that body composition and nutrient supply during these periods may impact fertility and early embryo viability (Shankar et al., 2008; Watkins et al., 2008; Lane et al., 2015; Stang and Huffman, 2016; Yang et al., 2021) as well as induce long-term metabolic effects on offspring (Satpathy et al., 2008; Smedts et al., 2008; Wax, 2009; Stephenson et al., 2018; Panchenko et al., 2019).

The placenta, of fetal origin, is a key nutritional sensor and target tissue for epigenetic modifications (Tarrade et al., 2015). The placenta is involved in maternal-fetal exchanges, metabolism, endocrinology, and immune pathways. Any disruption in its development may alter its structure and function, notably the nutrient supply from mother to fetus, which controls harmonious fetal growth and birth weight. Inadequate maternal nutritional environment is known to damage placental function through impaired gene expression, vascular regulation inducing perturbed hemodynamic, altered endocrine function, and inflammation (Maltepe and Fisher, 2015; Burton et al., 2016; Staud and Karahoda, 2018). Thus, sensitive to the maternal environment, the placenta acts as a programming agent (Burton and Fowden, 2012). In addition, placental response to the adverse environment was shown to differ between males and females, leading to a distinctive signature according to sex (Tarrade et al., 2013, 2015).

The liver plays a central role in the regulation of homeostasis in response to changes in nutrient and environmental inputs (Jones, 2016). In addition, non-alcoholic fatty liver disease has become a major public health issue; among patients, 20–30% will progress to much more serious non-alcoholic steatohepatitis (NASH) (Black et al., 2019) with potential developmental origins (Wesolowski et al., 2017). The liver undergoes several metabolic transitions from the fetal-neonatal period to adulthood, with crucial windows, such as birth and weaning, characterized by marked nutritional changes, inducing transcriptional and metabolic adaptations (Bowman et al., 2019). So far, the programming of hepatic metabolism by maternal pre-natal or even pre-conceptional nutritional condition has been poorly studied (Safi-Stibler et al., 2020).

The objectives of this study were to discriminate between pre-conception and gestational effects of maternal high-fat diet on the feto-placental phenotype at 28 dpc (near term) in a rabbit model. This model was chosen because of its interest as a model for human reproduction (Fischer et al., 2012) and because its gestational response to this high-fat diet has already been described (Picone et al., 2011; Tarrade et al., 2013).

## Materials and Methods

### Bioethics

The animal study was reviewed and approved under N°11/037 by the local ethical committee for animal experimentation COMETHEA (“*Comité d’Ethique en Expérimentation Animale du Centre INRAE de Jouy en Josas et AgroParisTech*”), referenced as N°C2EA-45 in the French National registry CNREEA (“*Comité National de Réflexion Ethique sur l’Expérimentation Animale*”).

### Animals and Experimental Procedures

Female New Zealand white rabbits of INRA 1077 line were used. They were housed individually in the same facilities with temperature and light controlled environment. At 10 weeks of age, the does were divided into two groups, H and C that were fed *ad libitum* with either an 8%-lipid (including 6% of soybean oil)-cholesterol (0.2%)-enriched diet (high fat-high cholesterol diet, named to simplify HD as high-fat diet) or a control diet (CD, 2% of lipids), respectively. The nutrient and chemical compositions of CD and HD have been described previously (Picone et al., 2011; Tarrade et al., 2013). Briefly, HD contained quantitatively more fatty acids (FAs), mainly n-6 polyunsaturated fatty acids (PUFAs), namely linoleic acid (Supplementary Table 1 and Supplementary Figure 1), and provided 16% more energy than the control diet. At 18 weeks of age, the does (H: *n* = 20 females, C: *n* = 20 females) were superovulated by five subcutaneous administrations of pFSH (Stimufol^®^; Merial, Boehringer Ingelhein, Germany) at 12-h intervals, as described previously (Mamo et al., 2008; Reis e Silva et al., 2012), followed 12 h later by an intravenous administration of 30 IU HCG (Chorulon; Intervet MSD Santé Animale, Beaucouze, France) at the time of natural mating. One day post coitum (dpc), the does were euthanized (named embryo donors), and 1-day embryos were recovered from oviducts by flushing and collected in HEPES M199 at 37°C. In parallel, recipient H (rH, *n* = 10) or C (rC, *n* = 10) female rabbits previously synchronized with hCG (0.8 μg Receptal^®^; Intervet, France) were sedated with ketamin (Imalgen 1000^®^, 10 mg/kg) and anesthetized by inhalation using a mixture of isoflurane/oxygen (Rousseau-Ralliard et al., 2019), and embryo transfers were performed on female oviducts after laparotomy. Female rabbits are characterized by a duplex uterus, i.e., two separate functional uteri and cervices, opening into a vagina simplex. To avoid any recipient-induced effect, five to six embryos in the same developmental stage from H or C donors (dH and dC, respectively) were transferred, respectively to the right and left oviducts of H and C female recipients (rH and rC, respectively), leading to four groups of feto-placental units: dHrH, dHrC, dCrH, and dCrC, named to simplify HH (6 females and 6 males), HC(6 females and 9 males), CH (10 females and 10 males), and CC(5 females and 10 males), respectively; the initial letter refers to embryo condition.

**FIGURE 1:**
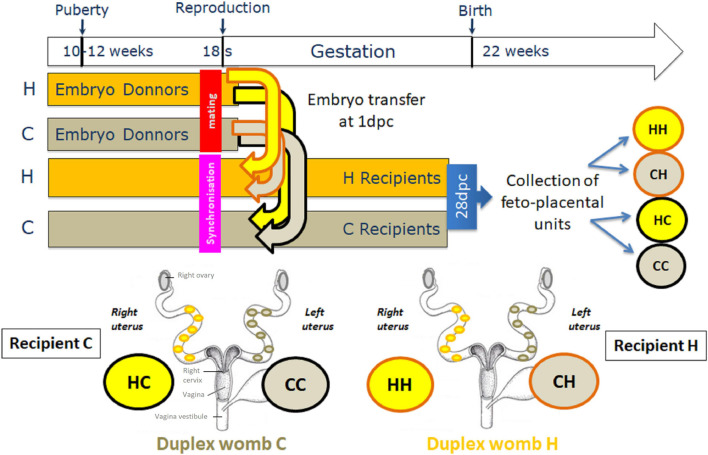
Experimental protocol. At 10 weeks of age, female New Zealand rabbits were fed *ad libitum* with either a high-fat high-cholesterol diet (HD) or a control diet (CD). Some of these females were super ovulated and then mated, and called embryo donors, while the others received hormone synchronization therapy, and called embryo recipients. Then, five to six 1-day embryos in the same developmental stage from H donors were transferred in the right horn, while five to six 1-day embryos from C donors were transferred in the left horn of each recipient C or H females, to avoid any substantial maternal-induced effect. Pregnancy continued in recipients until day 28 (3 days before term), at which point the feto-placental units were collected. Duplex uterus (womb) in rabbits is actually made up of two separate uteri, each with a cervix that opens into a single vagina.

Subsequently in the text, feto-placental samples from C or H embryos transferred in C recipient females are designated by CC and HC, and those from C or H embryos transferred in H recipient females are designated by CH and HH, respectively (Figure 1).

### Feto-Placental Collection 28 Days Post Coitum (28 dpc)

Twenty-eight dpc, i.e., 3 days before the end of the gestation, and following overnight fasting, after 30-min premedication with an analgesic (Torbugesic), laparotomy was performed on anesthetized (isoflurane) six H and five C recipient does, and four combinations of collected feto-placental units were obtained: CC (*n* = 15), CH (*n* = 15), HC (*n* = 20), and HH (*n* = 12). Fetal blood was obtained by ultrasound-guided intra-cardiac puncture, through the uterine wall of the mother prior to the collection of feto-placental units. A drop of fetal blood was immediately used to measure glycaemia using a glucose-strip-test (KM2902, FreeStyle Optium Xceed Medisense; ABBOTT, Chicago, United States). Blood samples were collected in EDTA-coated Vacutainers, then centrifuged, and plasma was stored at −20°C until analysis. The does were euthanized by lethal injection of Dolethal (5 ml; Vetoquinol, Lure, France). Fetuses, placentas, and deciduae were then immediately collected and weighed. Placentas from each group were dissected from the deciduae. Decidua is formed in a process called decidualization under the influence of progesterone. Decidualization is a process that occurs in early pregnancy, indeed, the maternal endometrial changes occur initially at the site of and following blastocyst implantation. Histologically, the placenta of rabbits is composed of the labyrinth zone, junctional zone, decidua zone of necrosis, decidua zone of separation, and mesometrium (Furukawa et al., 2014). The rabbit placental junctional zone is in contact with the maternal decidua. Indeed, because decidua and junctional area (fetal origin) are nested, it is, therefore, not possible to completely separate them by dissection (Lopez-Tello et al., 2019). The fetuses were euthanized by lethal injection of Dolethal and dissected. Kidneys, liver, heart, and the labyrinthine area of the placenta, and decidua, including the junctional zone, were weighed and either snap-frozen and stored at −80°C or transferred in chloroform-methanol (2/1, v/v) and stored at −20°C until further analyses.

### Sex Determination of Fetuses

Deoxyribonucleic acid was extracted from fetal liver, and PCR for determination of Y chromosome was performed as described elsewhere (Diaz-Hernandez et al., 2008).

### Transcriptomic Analyses

#### RNA Isolation

The liver and placental labyrinthine zone (50–100 mg) were reduced in 1 ml of TRizol^TM^ Reagent (15596; Thermo Fisher Scientific, CA, United States) with mixer Mill MM300 (Qiagen, Germantown, MD, United States) and one tungsten ball for 2 min at 20 Hz twice. Then, total RNA was extracted according to the instructions of the manufacturer (Life technologies, Thermo Fisher Scientific, CA, United States). After RNA precipitation with 500 μl of isopropanol and washing with 1 ml of 70% ethanol, total RNA was resuspended in RNAse free water and stored at −20°C. RNA concentration and purity (A260/A280 and A260/A230) were measured using a NanoDrop spectrophotometer (Nanodrop technology, Thermo Fisher Scientific, CA, United States). RNA degradation was checked by 1.5% agarose gel migration. RNA purification and DNase treatment were performed with ReliaPrep RNA tissue Miniprep Systems Kit (Z6110; Proméga, La Farlède, France). Another measure using the Nanodrop spectrophotometer was performed. Then, the quality of purified RNA was assessed using Bio-Analyzer Agilent 2100 (Agilent Biotechnologies). The RNA integrity number (RIN) of the samples was within the range of 7.3 and 10.

#### Reverse Transcription and Real-Time PCR

The RNA (800 ng) was reverse-transcribed using the iScript Reverse Transcription Supermix (Bio-Rad, Hercules, CA, United States). The expression of conventionally used housekeeping genes, such as RPL18 and EIF4E2, was shown to be stable between assays. Quantitative real-time PCR (qRT-PCR) was performed with the Fast SYBR Green Master Mix and the Applied Biosystems 7900HT Fast Real-Time PCR System (Applied Biosystems, Thermo Fisher Scientific, Foster City, CA, United States). The experimental protocol consisted of an initial polymerase activation at 95°C for 20 s followed by an amplification program for 40 cycles maintaining the annealing and extension primer temperature at 62°C for 20 s. A melting-curve analysis was then performed to verify the amplification of a single product. All primers were designed using NCBI Primer-BLAST and selected to generate amplicons with a length of 100–200 bp (Supplementary Table 2). The primers were synthesized by Eurogentec. Standard curves were generated to calculate the efficiency of each set. Only primer sets with an efficiency of 1.85–2.1 were used for qPCR. The relative mRNA levels for each assay were computed from the Ct values obtained for the target gene. The data were pooled and analyzed using RQ manager v1.2 and DataAssist v1.0 (Applied Biossytems, Thermo Fisher Scientific, CA, United States).

### Fatty Acid Profiling by Gas Chromatography

One hundred μl of plasma and samples of placental labyrinthine area (400 mg) and liver (200 mg) randomly chosen from each litter (to reach *n* = 6–8 per group and sex) were used for the analyses. Lipids were extracted with chloroform/methanol (2/1, V/V) (Folch et al., 1957). For placental and hepatic lipid extracts, phospholipid fraction, mostly representative of the membrane lipids, and neutral lipid fraction, representative of the storage lipids, were separated from non-phosphorous lipids on silica acid cartridges (Juaneda and Rocquelin, 1985). The fatty acids (FAs) from plasma lipids and tissue phospholipids and neutral lipids were transmethylated with Boron trifluoride methanol 7% (Sigma-Aldrich, Saint louis, United States) (Morrison and Smith, 1964). FA methyl esters were analyzed by gas chromatography coupled to FID (Gas Chromatograph 3900; Varian, Courtaboeurf, France) on an Econo-Cap EC-WAX capillary column (Tarrade et al., 2013). The FA profile was established for each sample with FA expressed as a percentage of total FAs. The analyses identified FA from C14:0 to C24:1n-9. In result tables, only a few relevant FAs are presented, but all of the identified FAs were considered in the calculation of total FAs. Heptadecanoic acid (C17:0, margaric acid), introduced prior to transmethylation, was used as an internal standard to obtain the relative quantification of total FAs, expressed either as mg/ml of plasma or mg/g of placenta or liver. The desaturase activity indices (DAIs) were estimated as the product:precursor ratios of individual FAs according to the following equations: Δ9 = [16:1ω7/16:0] and [18:1ω9/18:0], Δ6 = [18:3ω6)/18:2ω6)], and Δ5 = [20:4ω6/20:3ω6] (Warensjo et al., 2006; Robbez Masson et al., 2008).

### Statistics

To compare the four groups (CC, CH, HC, and HH), non-parametric Kruskal-Wallis tests were performed, followed by a pairwise comparison by Dunn’s multiple comparisons test (GraphPad Prism 7). *P* < 0.05 was considered statistically significant. Data are expressed as: median (Q1; Q3). The first quartile (Q1) and the third quartile (Q3) correspond to 25 and 75% percent of scores, respectively.

For lipid analyses on placenta, liver, and plasma, principal component analyses (PCAs) were additionally performed using the R statistical software (R Core Team, 2013).^1^ The FactomineR package (dedicated to multivariate data analysis; Lê et al., 2008) was included in R commander to analyze the biological relevance of observed FA changes, namely to extract important information and express this information as a set of new variables called principal components (Rousseau-Ralliard et al., 2019). These principal components can be used to visualize graphically the data. The individual’s factor map is a plot of the principal component scores for individuals on the first two principal components. The correlation circles that do not overlap sign significant differences between groups (significant v.test when an absolute value greater than 2). The variables factor map presents a view of the projection of the observed variables projected into the plane spanned by the first two principal components. Still named correlation circle, it can help to visualize the most correlated variables (i.e., variables that group together) and to show the structural relationship between the variables and the components. The projection of a variable vector onto the component axis allows us to directly read the correlation between the variable and the component. The cos^2^ values (and the v.test) are used to estimate the quality of the representation. The closer a variable is to the circle of correlations, the better its representation on the factor map (and the more important it is to interpret these components). Variables that are closed to the center of the plot are less important for the first components.

Multiple factor analysis is a multivariate data analysis method for summarizing and visualizing a complex data table in which individuals are described by several sets of variables (quantitative and/or qualitative) structured into groups, i.e., multiple factor analysis (MFA) is a global analysis where multiple sets of variables are simultaneously considered (De Tayrac et al., 2009). It takes into account the contribution of all active groups of variables to define the distance between individuals. The number of variables in each group may differ, and the nature of the variables (qualitative or quantitative) can vary from one group to the other, but the variables should be of the same nature in a given group. MFAs were performed on placenta data sets and liver data sets using the R statistical software and the package FactomineR.

## Results

### Biometrical Data

Data are expressed in Table 1. The HH fetuses were significantly growth retarded with decreased placental efficiency, compared to controls. They also had significantly lighter liver and kidneys compared to controls. Only fetal length was significantly increased in the CH fetuses compared to controls, but CH placental efficiency was decreased, with significantly heavier placenta and decidua. In contrast, absolute kidney weight was significantly reduced in CH compared to controls. There was no significant difference for any measurement between HC and controls.

**TABLE 1:** Impact of embryo origin and maternal diet during gestation on the biometry of feto-placetal units.

Groups⇒ Biometric data⇓	CC (*n* = 15)	dHrC (*n* = 15)	CH (*n* = 20)	HH (*n* = 12)	*P*-value	Significant differences between groups
Sex ratio (♀/♂)	5/10	6/9	10/10	6/6	–	–
Decidua (g)	1.70 [1.51; 2.01] a	1.73 [1.42; 2.00] a	2.19 [1.91; 2.34] b	1.86 [1.72; 2.08] ab	0.0029	(CC = HC) < (CH)
Placenta (g)	4.94 [4.51; 5.73] a	5.43 [5.00; 5.53] ab	6.19 [5.65; 6.82] b	4.79 [4.34; 6.31] ab	0.0091	(CC) < (CH)
Fetus BW (g)	37.8 [33.9; 37.4] a	35.9 [33.4; 38.1] a	32.5 [30.7; 36.8] ab	30.8 [25.8; 32.9] b	0.0082	(CC = HC) > (HH)
Fetal liver (g)	2.54 [2.45; 2.75] a	2.58 [2.13; 2.74] ab	2.67 [2.12; 2.94] ab	1.81 [1.61; 2.23] b	0.0067	(CC) > (HH)
Fetal liver to fetus BW ratio ×10^2^	7.6 [7.3; 7.9] ac	7.2 [6.3; 7.8] abc	7.0 [6.7; 8.7] ac	6.3 [6.0; 6.6] b	0.0093	(CC) > (HH) *et (CH) > (HH*)
Fetal kidneys (g)	0.35 [0.31; 0.36] a	0.36 [0.31; 0.38] a	0.30 [0.26; 0.34] b	0.23 [0.19; 0.30] b	0.0002	(CC = HC) > (CH = HH)
Fetal kidneys to fetus BW ratio ×10^3^	9.5 [8.9; 10.8] a	9.0 [8.1; 10.0] a	9.0 [8.4; 9.4] ab	8.5 [7.9; 9.1] b	0.0028	(CC = HC) > (HH)
Fetal length (cm)	9.0 [8.8; 9.3] a	9.2 [8.9; 9.4] a	11.6 [10.7; 12.1] b	9.3 [8.5; 11.3] a	<0.0001	(CC = HC = HH) < (CH)
Placental efficiency	5.1 [4.8; 5.7] a	4.9 [4.7; 5.4] ab	3.8 [3.5; 4.7] b	3.9 [3.6; 4.8] b	0.0010	(CC = HC) > (CH = HH)

*Statistics: Data were analyzed by Kruskal-Wallis test followed by, if applicable (i.e., if significant at p < 0.05), pairwise Dunn’s multiple comparisons test to explore pre-conception exposure (embryo origin) and in utero exposure (recipient diet during gestation). The data are represented by Median [Q1; Q3] as the median, the 1st and 3rd quartiles. Medians in a row without a common letter differ, P < 0.05.*

*CC, control embryo transferred in control recipient does; CH, control embryo transferred in HD-fed recipient does; HC, embryo from HD-fed donor transferred in control recipient does; HH, embryo from HD-fed donor transferred in HD-fed recipient does; ♀, females; ♂, males; BW, body weight; placental efficiency, fetus BW/placental weight ratio.*

### Fatty Acid Profiling and Concentrations

#### Decidua

**FIGURE 2:**
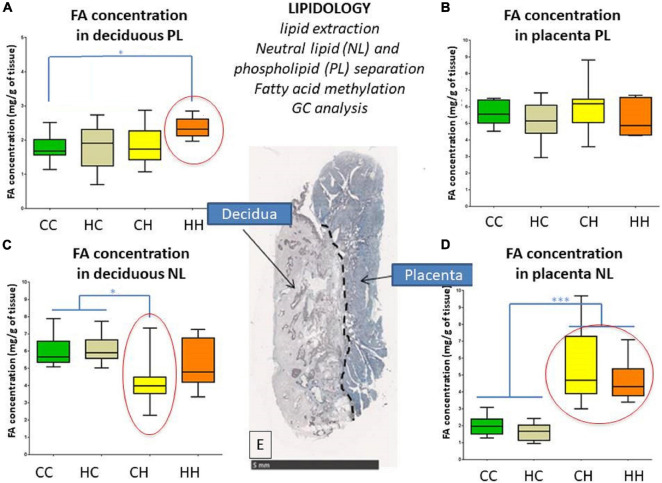
Impact of embryo origin and maternal diet during gestation on fatty acid (FA) concentrations in neutral lipid (NL, intracellular storage) and phospholipids (PLs, membranes) in decidua **(A,C)** and placenta **(B,D)** 28 days post coitum (dpc). **(A,C)** FA concentration in deciduous PL and NL; **(B,D)** FA concentration in placenta PL and NL; **(E)** histological section with classic HES staining showing on the left the deciduous and on the right the trophoblastic zones of the placenta. Arbitrary colors are assigned to each group just to improve group tracking. Legend of statistical analyses with *: *p* < 0.05 and ***: *p* < 0.001.

The FA concentrations in decidua phospholipids (i.e., membrane) were significantly higher (*p* = 0.034) in HH (2.25 [2.13; 2.51] mg/g of decidua) compared to CC (1.66 [1.49; 1.74] mg/g of decidua), while FA concentrations in the HC and CH groups were in between (with 1.92 [1.4; 2.23] and 1.72 [1.37; 2.34] mg/g of decidua, respectively) (Figure 2A).

The FA concentrations in decidua neutral lipids (i.e., intracellular storage) were significantly lower (*p* = 0.027) in CH (4.01 [3.48; 5.55] mg/g of decidua) compared to CC and HC (5.39 [5.21; 6.99] and 1.92 [1.4; 2.23] mg/g of decidua, respectively), whereas no difference was observed between HH and the other groups (Figure 2B).

The profiles of HC and CC deciduae were rather similar despite subtle differences when looking at the profile fatty acid by fatty acid (data not shown), suggesting that the origin of embryos was less important in the control recipient diet. The profile of the CH deciduae was closer to the that of CC than that of the HH, suggesting that the deciduae adapted to the fetus when it was healthy.

**FIGURE 3:**
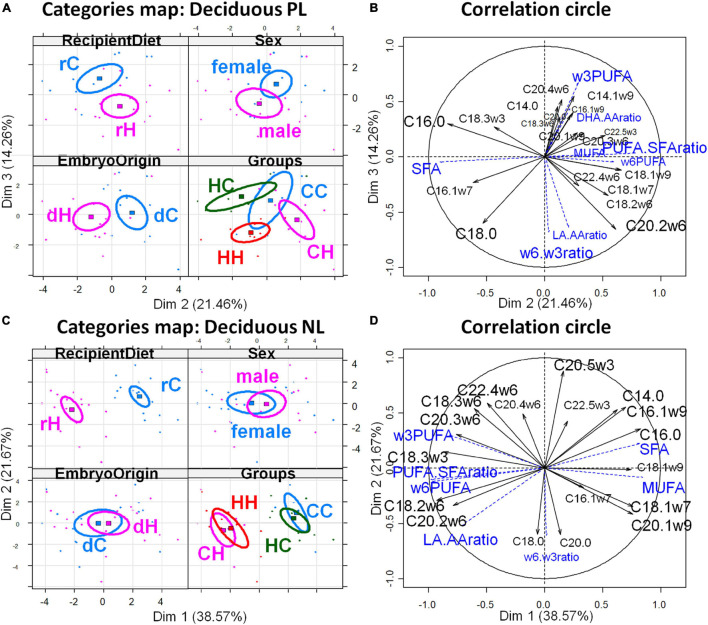
Principal component analyses of PL and NL FA profiles in deciduae 28 dpc, in response to embryo origin and gestational maternal diet. **(A)** Category map of deciduous PL. **(B)** Correlation circle of deciduous PL fatty acids. **(C)** Category map of deciduous NL. **(D)** Correlation circle of deciduous NL FAs. CC, control embryo transferred in control recipient does; CH, control embryo transferred in HD-fed recipient does; dC, C embryo donors; dH, H embryo donors; HC, embryo from HD-fed donor transferred in control recipient does; HH, embryo from HD-fed donor transferred in HD-fed recipient does. LA.AA ratio, linoleic-to-arachidonic acid ratio; MUFA: monounsaturated fatty acids; NL, neutral lipids correspond to intracellular lipid stores; PL, phospholipids correspond to membrane lipids; PUFA, polyunsaturated fatty acids; SFA, saturated fatty acids; rC, C recipient does; rH, H recipient does. Regarding decidua PL, maternal diet had weak effect, whatever the exposure period, since differences among the HC, CH, and HH groups appeared only on the second and third dimension while CC stood out from the other groups on dimension 5. Dimension 2 correlated with embryo origin [cos^2^ = 0.819, | v.test| = 3.363 positively with dC and negatively with dH (significant v.test when its absolute value is greater than 2)], the variables PUFA-to-SFA ratio, C22:4ω6, and C20:2ω6, as well as the CH group contribute positively, while the variables SFA and C16:0 et C16:1ω7, besides the HC group, contributed negatively to dimension 2. In contrast, dimension 3 correlated with the recipient diet (cos^2^ = 0.465, | v.test| = 3.101, positively with rC and negatively with rH), the variables ω3PUFA, C14:1ω9, AA, C14:0, as well as the HC group, contributed positively while ω6-to-ω3 PUFA ratio, C20:2ω6, LA-to-AA ratio, C18:0, LA, as well as the HH group contributed negatively to dimension 3. CC correlated on dimension 5 with DHA-to-AA ratio and C20:0 and C20:1. Regarding deciduae NL FA profiles, dimension 1 correlated very significantly and only with the diet of recipients (cos^2^ = 0.915, | v.test| = 5.39, positively with rC and negatively with rH), with rH represented by high ω6 PUFA (LA and C20:2ω6), LA-to-AA ratio, and PUFA-to-SFA ratio. The CC and HC groups, as well as CH and HH, were combined.

The PCAs of the decidual fatty acid profiles are shown in Figure 3 and confirm the above conclusions. The decidua membranes (PL) appeared weakly affected by the maternal diet, whatever the exposure period, since the differences among the HC, CH, and HH groups appeared only on the 2nd and 3rd dimensions, while CC stood out from the other groups on dimension 5. Dimension 2 correlated with embryo origin [cos^2^ = 0.819, | v.test| = 3.363 positively with dC and negatively with dH (significant v.test when its absolute value is greater than 2)] (Figure 3A); the variables PUFA-to-SFA ratio, C22:4ω6, and C20:2ω6, as well as the CH group contribute positively, while the variables SFA and C16:0 et C16:1ω7, besides the HC, group contributed negatively to dimension 2 (Figure 3B). In contrast, dimension 3 correlated with the recipient diets (cos^2^ = 0.465, | v.test| = 3.101, positively with rC and negatively with rH), the variables ω3PUFA, C14:1ω9, AA, C14:0, as well as the HC group contributed positively, while ω6-to-ω3 PUFA ratio, C20:2ω6, LA-to-AA ratio, C18:0, LA, as well as the HH group contributed negatively to dimension 3. CC correlated to dimension 5, along with DHA-to-AA ratio and C20:0 and C20:1. Regarding deciduae NL FA profiles, dimension 1 correlated very significantly and only with the diet of recipients (cos^2^ = 0.915, | v.test| = 5.39, positively with rC and negatively with rH) (Figure 3C), with rH represented by high ω6 PUFA (LA and C20:2ω6), LA-to-AA ratio, and PUFA-to-SFA ratio. The CC and HC groups, as well as CH and HH, were combined (Figure 3D).

#### Placenta (Labyrinthine Area, Fetal Origin)

For placental phospholipids (i.e., membranes), no significant change in FA concentrations was observed among the groups, with 5.55 [5.07; 6.31], 5.14 [4.66; 5.98], 6.18 [5.15; 6.44], and 4.88 [4.35; 6.2] mg/g of placenta in the CC, HC, CH, and HH groups, respectively (Figure 2C).

For neutral lipids (i.e., intracellular storage) in the placenta, FA concentrations were significantly different (*p* < 0.0001) according to recipient nutrition. For C recipients, concentrations were 1.97 [1.52; 2.34] and 1.67 [1.28; 2.03] mg/g of placenta in the CC and HC groups, respectively, whereas for H recipients, they went up to 4.67 [3.84; 7.13] and 4.19 [3.8; 5.21] mg/g of placenta in the CH and HH groups, respectively (Figure 2D).

**FIGURE 4:**
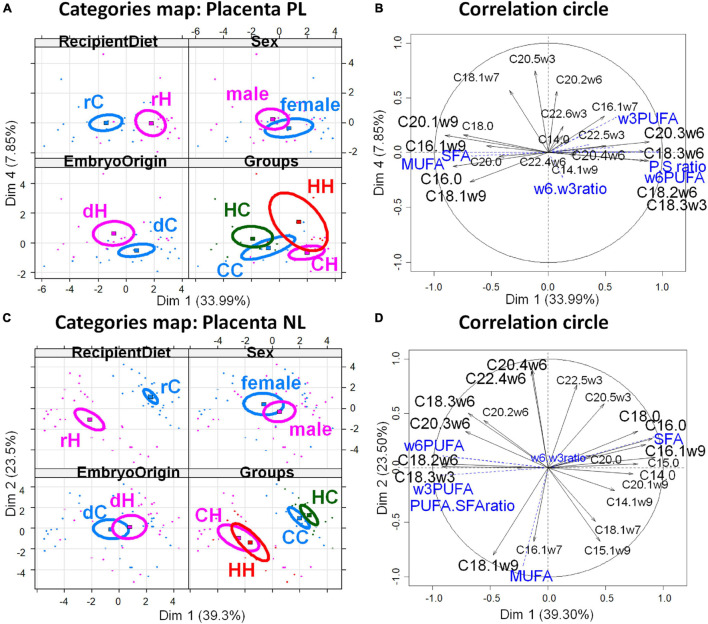
Principal component analyses of PL and NL FA profiles in placenta 28 dpc, in response to embryo origin and gestational maternal diet. **(A)** Category map of deciduous PL. **(B)** Correlation circle of deciduous PL fatty acids. **(C)** Category map of deciduous NL. **(D)** Correlation circle of deciduous NL FAs. CC, control embryo transferred in control recipient does; CH, control embryo transferred in HD-fed recipient does; dC, C embryo donors; dH, H embryo donors; HC, embryo from HD-fed donor transferred in control recipient does; HH, embryo from HD-fed donor transferred in HD-fed recipient does. LA.AA ratio, linoleic-to-arachidonic acid ratio; MUFA, monounsaturated fatty acids; NL, neutral lipids correspond to intracellular lipid stores; PL, phospholipids correspond to membrane lipids; PUFA, polyunsaturated fatty acids; SFA, saturated fatty acids; rC, C recipient does; rH, H recipient does. Regarding placenta PL, recipient diet correlated to dimension 1 (cos^2^ = 0.551, | v.test| = 3.912 positively with rH and negatively with rC) to which PUFA-to-SFA ratio and ω6 PUFA positively contributed while SFA and MUFA negatively contributed. In contrast, embryo origin correlated slightly to dimension 4 (cos^2^ = 0.215, | v.test| = 2.924 positively with dH and negatively with dC). Regarding placental NL, the CC and HC groups, as well as CH and HH, were either very close or combined, as shown by the correlation circles. Only the recipient diet had an effect. The FA profiles were highly represented by dimension 1 that was very significantly correlated with the diet of recipients (cos^2^ = 0.812, | v.test| = 6.139, positively with rC and negatively with rH), with rH represented by high PUFA-to-SFA ratio, C18:2ω6 (linoleic acid), and ω6 PUFA.

The PCA of FA profiles in placental PL demonstrated a strong impact of the maternal high-fat diet during gestation (recipient diet) on the CH and HH placentas with high concentrations of FA, more ω6 polyunsaturated FA (PUFA), and less monounsaturated FA (MUFA), than on CC and HC (Figures 4A,B). Thus, recipient diet correlated to dimension 1 (cos^2^ = 0.551, | v.test| = 3.912 positively with rH and negatively with rC) to which PUFA-to-SFA ratio and ω6 PUFA positively contributed, while SFA and MUFA negatively contributed. In contrast embryo origin correlated slightly to dimension 4 (cos^2^ = 0.215, | v.test| = 2.924 positively with dH and negatively with dC). Placental HC profiles differed from CC (ω6 PUFA deficiency and more saturated). The CH placentas presented more alteration in FA profiles than those of HH, with more MUFA. Altogether, the fatty acid profiles of neutral placental lipids reflected the maternal diets of recipients during gestation (Table 2).

**TABLE 2:** Fatty acid profiles (% of total fatty acids) of neutral lipid (intracellular storage) and phospholipids (membranes) in placenta 28 days post coitum (dpc), in response to embryo origin and gestational maternal diet.

Groups Fatty acids⇓	CC (*n* = 15)	HC (*n* = 15)	CH (*n* = 20)	HH (*n* = 12)	*P*-value	Significant differences between groups
**Placenta phospholipids**						
C18:2 ω6 (LA)	15.4 [14.1; 16.8] a	11.4 [10.8; 12.2] b	17.4 [15.8; 19.3] a	16.5 [16.4; 16.7] a	0.0020	(HC) < (CC = CH = HH)
C18:3 ω3 (ALA)	0.10 [0.06; 0.11] a	0.07 [0.05; 0.09] a	0.15 [0.12; 0.19] b	0.13 [0.11; 0.17] b	<0.0001	(CC = HC) < (CH = HH)
C20:3 ω6	4.8 [3.5; 5.5] a	4.1 [3.9; 4.4] a	6.7 [6.5; 7.0] b	6.3 [5.8; 7.2] b	<0.0001	(CC = HC) < (CH = HH)
C22:6 ω3	0.20 [0.14; 0.27] a	0.21 [0.13; 0.37] a	0.12 [0.09; 0.15] b	0.17 [0.14; 0.21] b	0.1423	ns
SFA	50.0 [49.0; 50.4] a	52.9 [50.6; 55.6] a	48.6 [47.4; 49.6] b	47.7 [46.4; 48.9] b	0.0020	(CC = HC) > (CH = HH)
MUFA	24.2 [23.5; 25.0] a	25.4 [23.8; 26.6] a	21.7 [20.9; 22.9] b	23.0 [22.0; 24.3] ab	0.0006	(CC = HC) > (CH)
ω6 PUFA	25.2 [24.0; 25.5] ab	20.6 [18.2; 23.1] a	29.3 [27.9; 30.8] b	27.7 [26.3; 29.3] b	0.0001	(HC) > (CH = HH)
ω3 PUFA	0.9 [0.7; 1.0]	0.9 [0.7; 1.2]	0.8 [0.7; 0.8]	0.9 [0.7; 0.9]	0.6280	ns
ω6/ω3 PUFA ratio	28 [26; 32] a	24 [19; 26] a	39 [34; 44] b	33 [31; 34] ab	<0.0001	(CC = HC) > (CH)
PUFA/SFA ratio	0.53 [0.49; 0.54] ab	0.41 [0.33; 0.47] a	0.62 [0.58; 0.67] b	0.61 [0.55; 0.65] b	0.0002	(HC) > (CH = HH)
Δ9 DAI (C16) ×10^2^	3.66 [3.27; 4.06]	3.42 [3.16; 3.68]	4.41 [3.16; 5.25]	5.34 [4.79; 6.21]	0.0029	(CC = HC) < (HH)
Δ6 DAI ×10^2^	4.19 [3.68; 5.16]	4.34 [4.05; 5.11]	5.28 [4.02; 6.72]	5.33 [4.69; 7.23]	0.1687	ns
Δ5 DAI	1.78 [1.64; 1.91]	1.75 [1.38; 2.06]	3.06 [2.83; 3.20]	2.79 [2.56; 3.09]	<0.0001	(CC = HC) < (CH = HH)
**Placenta neutral lipids**						
C18:2 ω6 (LA)	8.6 [6.4; 9.7] a	8.4 [7.7; 9.0] a	15.6 [11.3; 22.9] b	12.0 [20.2; 15.5] b	<0.0001	(CC = HC) < (CH = HH)
C18:3 ω3 (ALA)	0.30 [0.24; 0.46] a	0.33 [0.26; 0.29] a	0.92 [0.61; 1.62] b	0.72 [0.27; 0.87] ab	0.0007	(CC = HC) < (CH)
C20:4 ω6 (AA)	0.90 [0.57; 1.28]	0.98 [0.56; 1.19]	0.62 [0.45; 0.73]	0.61 [0.26; 0.92]	0.1029	ns
C20:5 ω3 (EPA)	0.11 [0.10; 0.18] a	0.19 [0.14; 0.25] a	0.07 [0.05; 0.09] b	0.05 [0.04; 0.09] b	<0.0001	(CC = HC) > (CH = HH)
SFA	53.7 [48.4; 55.8] a	53.5 [51.9; 54.8] a	30.1 [24.0; 33.7] b	32.0 [29.5; 35.0] b	<0.0001	(CC = HC) > (CH = HH)
MUFA	32.6 [31.5; 33.9] a	31.8 [30.6; 33.5] a	40.7 [37.2; 44.0] b	43.1 [37.9; 47.9] b	<0.0001	(CC = HC) < (CH = HH)
ω6 PUFA	14.0 [11.4; 16.3] a	13.6 [12.0; 16.0] a	32.7 [24.5; 35.0] b	25.6 [19.83; 31.4] b	<0.0001	(CC = HC) > (CH = HH)
ω3 PUFA	0.82 [0.59; 1.07] a	0.76 [0.58; 0.83] a	2.03 [1.06; 2.44] b	0.90 [0.43; 1.52] ab	0.0011	(CC = HC) < (CH)
ω6/ω3 PUFA ratio	18 [11; 23]	19 [17; 20]	18 [12; 23]	21 [16; 28]	0.5270	ns
PUFA/SFA ratio	0.29 [0.22; 0.32] a	0.27 [0.22; 0.32] a	1.23 [0.83; 1.51] b	0.79 [0.59; 1.18] b	<0.0001	(CC = HC) < (CH = HH)

*Statistics: Data were analyzed by Kruskal-Wallis test followed by, if applicable (i.e., if significant at p < 0.05), pairwise Dunn’s multiple comparisons test to explore pre-conception exposure (embryo origin) and in utero exposure (recipient diet during gestation). The data are represented by Median [Q1; Q3] as the median, the 1st and 3rd quartiles. Medians in a row without a common letter are significantly different, P < 0.05.*

*CC, control embryo transferred in control recipient does; CH, control embryo transferred in HD-fed recipient does; HC, embryo from HD-fed donor transferred in control recipient does; HH, embryo from HD-fed donor transferred in HD-fed recipient does;. ALA, alpha linolenic acid; AA, arachidonic acid; DAI, desaturase activity indices; EPA, eicosapentaenoic acid; LA, linoleic acid; MUFA, monounsaturated fatty acids; PUFA, polyunsaturated fatty acids; SFA, saturated fatty acids.*

Regarding placental NL, the CC and HC groups, as well as CH and HH, were either very close or combined, as shown by the correlation circles (Figure 4C). Only the recipient diet had an effect. The FA profiles were highly represented by dimension 1 that was very significantly correlated with the diet of recipients (cos^2^ = 0.812, | v.test| = 6.139, positively with rC and negatively with rH), with rH represented by high PUFA-to-SFA ratio, C18:2ω6 (linoleic acid), and ω6 PUFA (Figure 4D).

#### Plasma

Fetal plasma fatty acids have been evaluated, but not enough samples from female fetuses could be obtained, so only data from males are presented. However, in a previous study, we have seen that in response to high fat-fed mothers, fetal plasma fatty acid concentration increased in a sex-specific manner, with higher concentration in males only (Tarrade et al., 2013).

**FIGURE 5:**
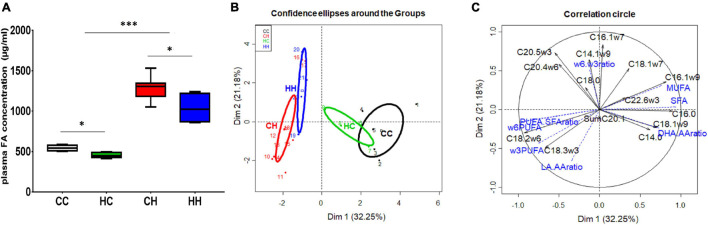
Fetal plasma FA analyses in male fetuses 28 dpc in response to embryo origin and gestational maternal diet. **(A)** Plasma fatty acid concentration. **(B)** Category map of groups. **(C)** Correlation circle of plasma FAs in male fetuses. CC, control embryo transferred in control recipient does; CH, control embryo transferred in HD-fed recipient does; dC, C embryo donors; dH, H embryo donors; HC, embryo from HD-fed donor transferred in control recipient does; HH, embryo from HD-fed donor transferred in HD-fed recipient does. AA, arachidonic acid (C204w6); DHA, docosahexaenoic acid (C22:6w3); DHA.AA ratio, docosahexaenoic-to-arachidonic acid ratio; LA.AA ratio, linoleic-to-arachidonic acid ratio; MUFA, monounsaturated fatty acids; PUFA, polyunsaturated fatty acids; SFA, saturated fatty acids. Legend of statistical analyses with *: *p* < 0.05 and ***: *p* < 0.001.

In the male fetuses, plasma FA concentrations were significantly higher (*p* = 0.0004) in the CH and HH groups (1,305 [1,210; 1,335] and 1,022 [888; 1,170] μg/ml of plasma, respectively) compared to the CC and HC groups (531 [521; 550] and 472 [460; 484] μg/ml of plasma, respectively), pointing out mainly the effect of the maternal diet during gestation (Figure 5A). In addition, concentrations in HC differed from that in CC (*p* = 0.016), and there was also a difference between CH and HH (*p* = 0.034).

Plasma FA profiles were also different, with a clear “recipient diet effect,” as observed for placenta (Table 3). Indeed, qualitative plasma FA profiles were similar between CC and HC, as shown by the overlapping correlation circles (Figure 5B), characterized by higher SFA and MUFA contents and higher DHA-to-AA ratio. Moreover, FA profiles were different in male fetuses collected from H recipients, depending on their embryo origin, as revealed by the separated correlation circles on the individual plot (Figure 5B). The qualitative plasma FA profile in CH was characterized by increased LA and ALA contents and higher LA-to-AA and PUFA-to-SFA ratios (Figure 5C), whereas the qualitative plasma FA profile in HH was characterized by higher contents in biologically active FAs, namely AA (C20:4ω6) and EPA (C20:5ω3).

**TABLE 3:** Impact of embryo origin and maternal diet during gestation on the plasma fatty acid profiles (% of total fatty acids) of male fetuses 28 dpc.

Groups Fatty acids⇓	CC (*n* = 5)	HC (*n* = 4)	CH (*n* = 9)	HH (*n* = 4)	*P*-value	Significant differences between groups
C14:0 (MA)	2.04 [2.02; 2.08] a	1.85 [1.83; 1.87] ab	1.56 [1.39; 1.67] b	1.52 [1.41; 1.59] b	0.0182	(CC) > (CH = HH)
C16:0 (PA)	36.2 [35.4; 36.7] a	36.0 [35.9; 36.0] ab	29.6 [29.0; 31.2] b	31.9 [31.5; 32.1] ab	0.0030	(CC) > (CH)
C18:0 (SA)	7.3 [6.8; 7.3]	6.9 [6.8; 6.9]	7.4 [7.1; 8.1]	7.7 [7.5; 7.7]	0.4169	ns
C18:1ω9 (OA)	19.9 [19.47; 20.5] a	20.1 [20.0; 20.2] a	16.7 [16.4; 16.8] b	16.1 [15.9; 16.3] b	0.012	(CC = HC) > (CH = HH)
C18:2 ω6 (LA)	18.5 [18.2; 18.7] a	18.4 [18.2; 18.7] ab	26.5 [24.7; 27.3] b	22.8 [22.4; 23.6] ab	0.0017	(CC) < (CH)
C18:3 ω3 (ALA)	1.06 [1.03; 1.12] ab	1.03[1.01; 1.04] a	1.35 [1.28; 1.67] b	1.20 [1.03; 1.38] ab	0.0051	(HC) < (CH)
C20:3 ω6	0.59 [0.54; 0.62] a	0.59 [0.58; 0.60] a	0.80 [0.75; 0.83] b	0.79 [0.72; 0.84] b	0.0016	(CC = HC) < (CH = HH)
C20:4 ω6 (AA)	3.63 [3.33; 3.91] a	3.96 [3.81; 4.10] ab	4.74 [4.10; 4.86] ab	5.15 [4.66; 5.46] b	0.0269	(CC) < (HH)
C20:5 ω3 (EPA)	0.055 [0.041; 0.059] a	0.057 [0.049; 0.065] ab	0.077 [0.075; 0.082] b	0.084 [0.079; 0.093] ab	0.0222	(CC) < (CH)
C22:6 ω3 (DHA)	0.25 [0.18; 0.28]	0.25 [0.24; 0.26]	0.18 [0.16; 0.20]	0.21 [0.19; 0.23]	0.3958	ns
SFA	45.9 [45.8; 46.4] a	45.7[45.6; 45.7] ab	39.1 [38.9; 40.8] b	41.8 [41.6; 41.9] ab	0.0011	(CC) > (CH)
MUFA	26.5 [26.4; 27.7] a	26.5 [26.5; 27.5] a	22.5 [21.5; 25.2] b	24.0 [23.4; 24.6] ab	0.0042	(CC = HC) > (CH)
ω6 PUFA	25.7 [24.5; 25.8] a	26.0 [26.0; 26.1] a	35.5 [34.3; 36.5] b	32.2 [31.6; 32.8] b	0.0006	(CC = HC) < (CH = HH)
ω3 PUFA	1.71 [1.64; 1.79] a	1.72 [1.70; 1.74] a	2.19 [2.01; 2.39] b	1.92 [1.71; 2.17] ab	0.0053	(CC = HC) < (CH = HH)
ω6/ω3 PUFA ratio	15 [14; 16]	15 [15; 15]	16 [15; 17]	17 [14; 18]	0.6311	ns
PUFA/SFA ratio	0.59 [0.57; 0.61] a	0.61 [0.61; 0.62] a	0.94 [0.90; 1.00] b	0.81 [0.79; 0.84] ab	0.0005	(CC = HC) < (CH)
LA/AA ratio	4.7 [3.9; 5.2]	4.7 [4.5; 4.9]	5.5 [4.7; 6.9]	4.5 [4.1; 5.1]	0.2050	ns
EPA/AA ratio×10^2^	1.506 [1.361; 1.545] a	1.568 [1.356; 1.879] ab	1.649 [1.596; 1.852] ab	1.966 [1.752; 2.164] b	0.0196	(CC) < (HH)
DHA/AA ratio	0.070 [0.063; 0.070] a	0.063 [0.063; 0.064] ab	0.039 [0.035; 0.048] b	0.044 [0.038; 0.049] ab	0.0049	(CC) > (CH)
Δ9 DAI (C16) ×10^2^	9.2 [8.8; 9.3] ab	9.0 [8.8; 9.2] (a)	10.2 [7.7; 10.7] ab	15.1 [12.8; 17.2] (b)	0.0868	ns
Δ6 DAI ×10^2^	11.1[11.1; 11.6]	12.9 [12.4; 13.5]	11.8 [11.7; 13.1]	11.9 [11.5; 12.4]	0.5825	ns
Δ5 DAI	16.4 [13.5; 17.0]	15.1 [14.5; 15.6]	16.9 [15.6; 19.5]	15.8 [14.9; 16.5]	0.1885	ns

*Statistics: Data were analyzed by Kruskal-Wallis test followed by, if applicable (i.e., if significant at p < 0.05), pairwise Dunn’s multiple comparisons test to explore preconception exposure (embryo origin) and in utero exposure (recipient diet during gestation). The data are represented by Median [Q1; Q3] as the median, the 1st and 3rd quartiles. Medians in a row without a common letter differ, P < 0.05.*

*CC, control embryo transferred in control recipient does; CH, control embryo transferred in HD-fed recipient does; HC, embryo from HD-fed donor transferred in control recipient does; HH, embryo from HD-fed donor transferred in HD-fed recipient does; AA, arachidonic acid; ALA, alpha linolenic acid; DAI, desaturase activity indices; DHA, docosahexaenoic acid; EPA, eicosapentaenoic acid; LA, linoleic acid; MA, myristic acid; MUFA, monounsaturated fatty acids; OA, oleic acid; PA, palmitic acid; PUFA, polyunsaturated fatty acids; SA, stearic acid; SFA, saturated fatty acids.*

#### Liver

FA concentrations in fetal liver phospholipids (PL, i.e., membranes) were significantly lower in the CH group (5.51 [5.48; 6.35] mg/g of fetal liver, *p* = 0.007) compared to groups raised by control recipients, namely CC and HC (6.51 [6.42; 6.63] and 6.45 [6.31; 7.02] mg/g of fetal liver, respectively), while FA concentration in the HH group (6.31 [6.09; 6.5] mg/g of fetal liver) remained intermediate.

FA concentrations in liver neutral lipids (NL, i.e., intracellular lipid storage) were not statistically different between groups (*p* = 0.81) with 33.2 [31.1; 39.9], 37.8 [36.1; 38.6], 35.5 [31; 43.5], and 34.9 [32.4; 52] mg FA/g of fetal liver in the CC, HC, CH, and HH groups, respectively.

**FIGURE 6:**
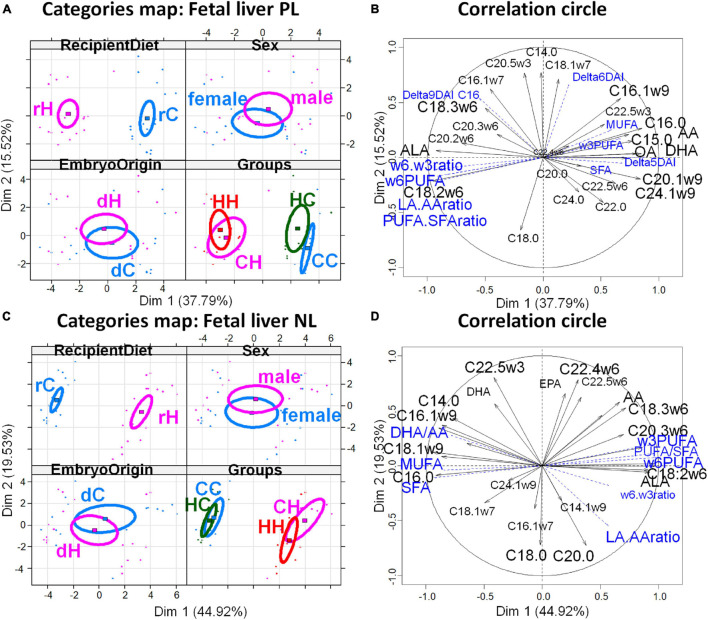
Principal component analyses of PL and NL FA profiles in fetal liver 28 dpc, in response to embryo origin and gestational maternal diet. **(A)** Category map of deciduous PL. **(B)** Correlation circle of deciduous PL FAs. **(C)** Category map of deciduous NL. **(D)** Correlation circle of deciduous NL fatty acids. CC, control embryo transferred in control recipient does; CH, control embryo transferred in HD-fed recipient does; dC, C embryo donors; dH, H embryo donors; HC, embryo from HD-fed donor transferred in control recipient does; HH, embryo from HD-fed donor transferred in HD-fed recipient does. AA, arachidonic acid (C204w6); ALA, alpha linolenic acid (C18:3w3); DAI, desaturase activity index; DHA, docosahexaenoic acid (C22:6w3); LA/AA ratio, linoleic-to-arachidonic acid ratio; MUFA, monounsaturated fatty acids; NL, neutral lipids correspond to intracellular lipid stores; OA, oleic acid (C18:1w9); PL, phospholipids correspond to membrane lipids; PUFA, polyunsaturated fatty acids; SFA, saturated fatty acids; rC, C recipient does; rH, H recipient does. Regarding fetal liver PL, maternal high-fat diet had a strong impact during gestation (recipient diet) on CH and HH placentas with higher content in ω6 PUFA and lower content in SFA compared to CC and HC. The recipient diet correlated to dimension 1 (cos^2^ = 0.965, | v.test| = 5.685 positively with rC and negatively with rH) to which SFA, C16:0, MUFA, delta5DAI, and DHA-to-AA ratio positively contributed, while LA, LA-to-AA ratio, and ω6 PUFA and ω6-to-ω3 PUFA ratio negatively contributed. In contrast, embryo origin correlated to dimension 3 (cos^2^ = 0.622, | v.test| = 2.791 positively with dC and negatively with dH). HC liver profiles differed from CC with more MUFA and increased delta5C16DAI and lower contents in C20:2 and C22:4ω6 PUFA, and long chain SFA (C20:0, C22:0, and C24:0). In contrast, fetal liver membrane profiles did not differ significantly between the CH and HH groups. Regarding fetal liver neutral lipids, they reflected the recipient diet with dimension 1 correlating very strongly with the diet of recipients (cos^2^ = 0.969, | v.test| = 5.608, positively with rH and negatively with rC), with rH represented by high ω6 PUFA, C18:2ω6 (LA), PUFA-to-SFA ratio, and ω6-to-ω3 PUFA and LA-to-AA ratios. The CC and HC groups, as well as CH and HH, were either very close or combined.

As previously seen on placental FA profiling, most of the differences between groups occurred in response to the recipient diets (Table 4). Thus, the FA profiles of both fetal liver NL and PL lipids reflected the recipient diet, with higher ω6 PUFA and LA in the HH and CH groups compared to the CC and HC groups, which, in contrast, presented higher MUFA and SFA contents. The PCA analysis (Figure 6), however, demonstrated some slight differences differentiating the groups, especially in membranes lipids (PL).

**TABLE 4:** Fatty acid profiles (% of total fatty acids) of neutral lipid (intracellular storage) and phospholipids (membranes) in fetal liver 28 dpc, in response to embryo origin and gestational maternal diet.

Groups Fatty acids⇓	CC (*n* = 15)	HC (*n* = 15)	CH (*n* = 20)	HH (*n* = 12)	*P*-value	Significant differences between groups
**Fetal liver phospholipids (PL)**						
C18:2 ω6 (LA)	19.7 [18.8; 20.2] a	19.4 [19.0; 20.2] a	25.3 [24.4; 26.4] a	24.6 [23.9; 26.1] a	<0.0001	(CC = HC) < (CH = HH)
C18:3 ω3 (ALA)	0.25 [0.24; 0.27] a	0.25 [0.23; 0.26] a	0.44 [0.41; 0.46] b	0.44 [0.42; 0.46] b	<0.0001	(CC = HC) < (CH = HH)
C20:4ω6 (AA)	9.7 [9.2; 10.2] a	9.2 [9.0; 9.7] ab	8.8 [8.4; 9.0] b	8.7 [8.7; 9.1] b	0.0052	(CC) > (CH = HH)
SFA	42.8 [42.4; 43.5] a	42.4 [42.1; 42.7] a	40.6 [40.1; 41.1] b	40.2 [39.9; 40.8] b	0.0002	(CC = HC) > (CH = HH)
MUFA	18.1 [17.5; 18.5] a	19.2 [18.7; 19.4] a	15.4 [14.9; 15.7] b	16.3 [16.0; 18.2] b	<0.0001	(CC = HC) > (CH = HH)
ω6 PUFA	35.1 [34.8; 36.6] a	35.2 [34.6; 35.5] a	40.7 [39.8; 41.8] b	39.8 [39.1; 40.6] b	<0.0001	(CC = HC) < (CH = HH)
ω3 PUFA	3.2 [2.9; 3.5] a	3.1 [2.9; 3.5] a	2.8 [2.5; 2.9] b	2.6 [2.5; 2.7] ab	0.0043	(CC = HC) > (CH)
ω6/ω3 PUFA ratio	11 [10; 12.2] a	11 [10; 12] a	15 [14; 16] b	16 [14; 16] b	<0.0001	(CC = HC) < (CH = HH)
PUFA/SFA ratio	0.90 [0.89; 0.92] a	0.90 [0.89; 0.91] a	1.07 [1.03; 1.09] b	1.05 [1.03; 1.07] b	<0.0001	(CC = HC) < (CH = HH)
Δ9 DAI (C16) ×10^2^	3.44 [3.27; 3.82] a	3.78 [3.72; 3.85] ab	3.74 [3.58; 4.69] b	5.94 [5.41; 10.05] c	0.0005	(CC) < (CH) < (HH)
Δ6 DAI ×10^2^	4.17 [3.86; 5.03]	4.23 [4.10; 6.20]	4.64 [4.26; 5.08]	4.59 [4.24; 4.95]	0.9051	ns
Δ5 DAI	5.08 [5.04; 5.61] a	4.78 [4.63; 5.36] ab	4.58 [4.15; 4.73] b	4.28 [4.16; 4.59] b	0.0015	(CC) > (CH = HH)
**Fetal liver neutral lipids (NL)**						
C18:2 ω6 (LA)	22.3 [21.9; 22.7] a	21.2 [20.5; 22.4] a	34.8 [32.8; 35.4] b	30.1 [28.2; 31.5] b	<0.0001	(CC = HC) < (CH = HH)
C18:3 ω3 (ALA)	2.01 [1.97; 2.04] a	1.85 [1.81; 2.04] a	2.71 [2.61; 2.85] b	2.55 [2.50; 2.62] ab	<0.0001	(CC = HC) < (CH = HH)
C20:4 ω6 (AA)	0.91 [0.89; 1.04] ab	0.91 [0.86; 0.97] a	1.18 [1.12; 1.24] b	1.01 [0.97; 1.09] ab	0.0013	(HC) < (CH)
C22:6ω3 (DHA)	0.065 [0.060; 0.079] ab	0.070 [0.061; 0.074] a	0.046 [0.042; 0.050] b	0.045 [0.040; 0.053] ab	0.0077	(HC) > (CH)
SFA	38.3 [37.0; 39.0] a	39.4 [36.2; 39.7] a	29.3 [27.8; 31.9] b	31.8 [31.1; 32.8] b	<0.0001	(CC = HC) > (CH = HH)
MUFA	32.4 [31.5; 33.3] a	31.8 [30.6; 33.5] a	40.7 [37.2; 44.0] b	43.1 [37.9; 47.9] b	<0.0001	(CC = HC) < (CH = HH)
ω6 PUFA	26.7 [26.4; 27.5] a	25.5 [24.7; 27.0] a	40.7 [38.5; 41.5] b	35.5 [33.1; 36.8] b	<0.0001	(CC = HC) < (CH = HH)
ω3 PUFA	2.75 [2.62; 2.92] a	2.58 [2.47; 3.01] a	3.58 [3.37; 3.77] b	3.37 [3.28; 3.55] b	<0.0001	(CC = HC) < (CH = HH)
ω6/ω3 PUFA ratio	9.7 [9.2; 10.1] a	9.8 [8.80; 10.1] a	11.0 [10.7; 11.4] b	10.3 [10.0; 10.7] ab	0.0030	(CC = HC) < (CH)
PUFA/SFA ratio	0.78 [0.74; 0.82] a	0.71 [0.68; 0.84] a	1.54 [1.30; 1.59] b	1.19 [1.12; 1.29] b	<0.0001	(CC = HC) < (CH = HH)

*Statistics: Data were analyzed by Kruskal-Wallis test followed by, if applicable (i.e., if significant at p < 0.05), pairwise Dunn’s multiple comparisons test to explore pre-conception exposure (embryo origin) and in utero exposure (recipient diet during gestation). The data are represented by Median [Q1; Q3] as the median, the 1st and 3rd quartiles. Medians in a row without a common letter differ, P < 0.05.*

*CC, control embryo transferred in control recipient does; CH, control embryo transferred in HD-fed recipient does; HC, embryo from HD-fed donor transferred in control recipient does; HH, embryo from HD-fed donor transferred in HD-fed recipient does; AA, arachidonic acid; ALA, alpha linolenic acid; DAI, desaturase activity indices; DHA, docosahexaenoic acid; LA, linoleic acid; MUFA, monounsaturated fatty acids; PUFA, polyunsaturated fatty acids; SFA, saturated fatty acids.*

The PCA of FA profiles in fetal liver PL revealed a strong impact of the maternal high-fat diet during gestation (recipient diet) on the CH and HH placentas with higher content in ω6 PUFA and lower content in SFA compared to CC and HC. The recipient diet correlated to dimension 1 (cos^2^ = 0.965, | v.test| = 5.685 positively with rC and negatively with rH) to which SFA, C16:0, MUFA, delta5DAI, and DHA-to-AA ratio positively contributed, while LA, LA-to-AA ratio, ω6 PUFA, and ω6-to-ω3 PUFA ratio negatively contributed. In contrast, embryo origin correlated to dimension 3 (cos^2^ = 0.622, | v.test| = 2.791 positively with dC and negatively with dH) (Figure 6B). HC liver profiles differed from CC with more MUFA and increased delta5C16DAI and lower contents in C20:2 and C22:4ω6 PUFA and long chain SFA (C20:0, C22:0, and C24:0) (Figure 6A). In contrast, fetal liver membrane profiles did not differ significantly between the CH and HH groups.

The FA profiles of neutral fetal liver lipids reflected the recipient diet (Table 4). For fetal liver NL FA profiles, dimension 1 correlated very strongly with the diet of recipients (cos^2^ = 0.969, | v.test| = 5.608, positively with rH and negatively with rC), with rH represented by high ω6 PUFA, C18:2ω6 (linoleic acid, LA), PUFA-to-SFA ratio, and ω6-to-ω3 PUFA and LA-to-AA ratios (Figure 6D). The CC and HC groups, as well as CH and HH, were either very close or combined (Figure 6C).

### Target Gene Expression in Placenta and Fetal Liver

#### Placenta Gene Expression

The IUGR and dyslipidemia observed in fetuses carried by recipient H mother, i.e., from the CH and HH groups, together with high placental accumulation of lipids, strongly suggested that the expression of placental genes involved in nutrient transfer and metabolism could be altered. Consequently, the expression of a selected number of relevant genes involved in trans-placental transfer was quantified by RT-qPCR (Table 5).

**TABLE 5:** Impact of embryo origin and maternal diet during gestation on the expression of genes involved in metabolism and nutrient transfer in placenta 28 dpc.

Recipient diet	Control (C)	Control (C)	High fat (H)	High fat (H)	Kruskal-Wallis test	Dunn’s *post-hoc* comparison test
Embryo origin	dC	dH	dC	dH		
Groups Targeted genes⇓	CC (*n* = 16)	HC (*n* = 16)	CH (*n* = 20)	HH (*n* = 12)	*P*-value	Significant differences between groups
Rabbit ABC-A1	0.27 [0.23; 0.28] a	0.29 [0.25; 0.32] a	0.08 [0.05; 0.12] b	0.14 [0.04; 0.17] b	<0.0001	(CC = HC) > (CH = HH)
Rabbit ABC-G1	0.15 [0.13; 0.19] a	0.16 [0.13; 0.22] a	0.04 [0.02; 0.05] b	0.05 [0.01; 0.08] b	<0.0001	(CC = HC) > (CH = HH)
Rabbit Adipo	0.45 [0.41; 0.53] a	0.43 [0.35; 0.65] a	0.99 [0.60; 1.60] b	0.99 [0.55; 1.13] b	<0.0001	(CC = HC) < (CH = HH)
Rabbit CD36	17.3 [14.8; 20.2]	16.2 [15.4; 18.0]	23.3 [17.3; 30.8]	16.8 [12.1; 18.9]	0.0606	ns
Rabbit FAS	0.076 [0.065; 0.095] a	0.093 [0.064; 0.098] a	0.010 [0.004; 0.015] b	0.008 [0.006; 0.020] b	<0.0001	(CC = HC) > (CH = HH)
Rabbit FATP4	0.020 [0.015; 0.022] a	0.020 [0.018; 0.024] a	0.010 [0.004; 0.014] b	0.007 [0.005; 0.011] b	<0.0001	(CC = HC) > (CH = HH)
Rabbit HMG CoA	0.13 [0.11; 0.16] ab	0.16 [0.15; 0.20] a	0.12 [0.07; 0.15] b	0.11 [0.09; 0.14] b	0.0005	(CC = HC) > (CH = HH)
Rabbit LDL-R	1.51 [1.12; 2.26] a	1.57 [0.70; 2.11] a	0.42 [0.32; 0.76] b	0.54 [0.06; 0.71] b	<0.0001	(CC = HC) > (CH = HH)
Rabbit LXR α	0.80 [0.48; 0.83]	0.72 [0.33; 0.84]	0.56 [0.41; 0.70]	0.62 [0.12; 0.78]	0.4536	ns
Rabbit PPARγ	0.27 [0.23; 0.28]	0.29 [0.25; 0.32]	0.08 [0.05; 0.12]	0.14 [0.04; 0.17]	0.0741	ns
Rabbit RXR α	0.058 [0.048; 0.080] a	0.053 [0.045; 0.079] a	0.024 [0.015; 0.038] b	0.022 [0.016; 0.025] b	<0.0001	(CC = HC) > (CH = HH)
Rabbit SLC2A1	2.51 [1.94; 3.15] a	2.58 [2.36; 3.00] a	1.01 [0.64; 1.38] b	0.78 [0.20; 1.24] b	<0.0001	(CC = HC) > (CH = HH)
Rabbit SLC2A3	1.76 [1.52; 2.08] a	1.86 [1.65; 2.0] a	0.57 [0.46; 0.77] b	0.43 [0.29; 0.64] b	<0.0001	(CC = HC) > (CH = HH)
Rabbit SLC38A1	0.70 [0.61; 0.77]	0.72 [0.68; 0.80]	0.63 [0.42; 0.82]	0.56 [0.13; 0.77]	0.1722	ns
Rabbit SLC38A2	1.09 [1.03; 1.26] a	1.34 [1.12; 1.42] a	0.58 [0.35; 0.91] b	0.69 [0.15; 0.95] b	<0.0001	(CC = HC) > (CH = HH)
Rabbit SLC38A4	0.016 [0.014; 0.020]	0.020 [0.017; 0.026]	0.013 [0.010; 0.018]	0.011 [0.009; 0.042]	0.0616	ns
Rabbit SREBP2	0.025 [0.021; 0.038]	0.026 [0.024; 0.033]	0.033 [0.021; 0.037]	0.045 [0.024; 0.057]	0.2295	ns

*Relative gene expression, obtained by RT-qPCR (2^–ΔCt^) of genes encoding ATP-binding cassette A1 and subfamily G member 1 for the transport of cholesterol (ABC-A1 and ABC-G1), adipophilin (Adipo), differentiation cluster glycoprotein 36 (CD36), fatty acid synthase (FAS), fatty acid transport protein 4 (FATP4), 3-hydroxy-3-méthylglutaryl-coenzyme A(HMG CoA), low-density lipoprotein receptor (LDL-R), nuclear receptors Liver X receptors (LXRα), peroxisome proliferator-activated receptor gamma (PPARγ), retinoic X receptor alpha (RXRα), solute carrier family 2 (facilitated glucose transporter) member 1 known as GLUT-1 (SLC2A1), GLUT-3 (SLC2A3), sodium-coupled neutral amino acid transporters 1, 2, and 4 (SLC38A1, SLC38A2, and (SLC38A4), and sterol regulatory element-binding protein 2 (SREBP2) in the placenta 28 dpc. The values were normalized with DataAssists Software, using two reference genes coding for 4E translation initiation factor 2 (EIF4E2) and ribosomal protein L18 (RPL18). Statistically, Data were analyzed by Kruskal-Wallis test followed by, if applicable (i.e., if significant at p < 0.05), pairwise Dunn’s multiple comparisons test to explore pre-conception exposure (embryo origin) and in utero exposure (recipient diet during gestation). The data are represented by Median [Q1; Q3] as the median, the first and third quartiles. Medians in a row without a common letter differ, p < 0.05.*

*CC, control embryo transferred in control recipient does; CH, control embryo transferred in HD-fed recipient does; HC, embryo from HD-fed donor transferred in control recipient does; HH, embryo from HD-fed donor transferred in HD-fed recipient does.*

Glucose transporters: The gene expression of both glucose transporters, such as SLC2A1 (for GLUT1), and SLC2A3 (for GLUT4) was decreased in the CH and HH groups compared to CC and HC. Similarly, amino acid transporter SLC38A2 transcripts were reduced or, SLC38A1 and SLC38A4, tended to be reduced in the CH and HH groups compared to the CC and HC groups.

Genes involved in lipid and cholesterol metabolism: Adipophilin expression was increased in CH and HH, and the expression of CD36 tended to be increased in CH only. All the other studied genes involved in lipid metabolism and transport were downregulated in the CH and HH groups compared to CC and HC. Indeed, the expression of genes involved in cholesterol transporters (ABC-A1 and ABC-G1) and LDR receptor or cholesterol trafficking (HMG-CoA reductase) was decreased in CH and HH. Similarly, FA transporters (FATP4) or genes involved in lipid metabolism (FAS and PPARγ and its partner RXRα) were downregulated in CH and HH (Table 5).

**FIGURE 7:**
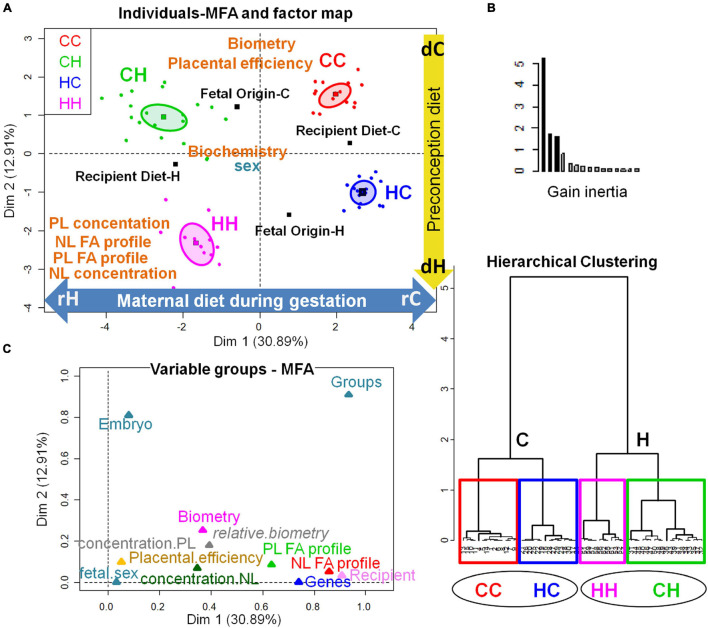
Multifactorial analysis of the combination of feto-placental biometry, placenta gene expression, and placenta FA signatures and concentrations. **(A)** Individuals-MFA and Factor map (with confidence ellipses around group categories). **(B)** Hierarchical clustering (with graph of inertia gain). **(C)** Variable groups-MFA.CC, embryo from control donor transferred in control recipient does; CH, embryo from control donor transferred in HD-fed recipient does; HC, embryo from HD-fed donor transferred in control recipient does; HH, embryo from HD-fed donor transferred in HD-fed recipient does; dC, control donor; dH, H embryo donors; rC, C recipient does; rH, H recipient does. The plot Variable groups-MFA illustrates the correlation between groups and dimensions. On the one hand, recipient diet correlated with dimension 1 (cos^2^ = 0.988, | v.test| = 7.438, positively with rC and negatively with rH) with a positive contribution of NL SFA and gene expression of nutrient transporters (namely SLC2A3 and A1, ABC A1 and G1, FAS, RXR, LDL-R, SLC38A2, and FATP4 among the genes analyzed) and a negative contribution of NL FA concentration, NL (and PL) w6 PUFA, NL linoleic and alpha-linolenic acid and PUFA-to-SFA ratio. On the other hand, fetal origin correlated with dimension 2 (cos^2^ = 0.772, | v.test| = 7.025, positively with dC and negatively with dH) with a positive contribution of biometry data (fetal, liver, and kidney weights), PL FA concentration and a negative contribution of NL and PL MUFA. The Lg coefficients of relationship between the contingency tables allow to measure to what extent the tables are related two by two. The more variables of a first table are related to the variables of the second table, the higher the Lg coefficient, this is represented by their proximity on the graph C.

A sex-specific effect could not be explored, because the n numbers of females and males were too small. Nevertheless, the multifactorial analysis (MFA) combining biometric data sets and placenta gene expression with placenta (NL and PL) fatty acid concentration and profiles showed that although the maternal diet during gestation played a preponderant role, a clear separation of the four groups was possible, either on the factor map (Figure 7A) or hierarchical clustering (Figure 7B). Thus, the HC group appeared completely dissociated from CC. The plot Variable groups-MFA illustrates the correlation between groups and dimensions (Figure 7C). On the one hand, recipient diet correlated with dimension 1, positively with rC corresponding to the positive contribution of NL SFA and gene expression of nutrient transporters (namely SLC2A3 and A1, ABC A1 and G1, FAS, RXR, LDL-R, SLC38A2, and FATP4 among the genes analyzed), and negatively with rH corresponding to the negative contribution of NL FA concentration, NL (and PL) w6 PUFA, NL linoleic and alpha-linolenic acid, and PUFA-to-SFA ratio. On the other hand, embryo origin correlated with dimension 2 positively with dC corresponding to the positive contribution of biometry data (fetal, liver, and kidney weights), and PL FA concentration, and negatively with dH corresponding to the negative contribution of NL and PL MUFA.

#### Fetal Liver Gene Expression

The IUGR and dyslipidemia observed in fetuses carried by recipient H mother, i.e., from the CH and HH groups, together with high hepatic accumulation of lipids in the CH and HH fetuses, strongly suggested that the expression of fetal liver genes involved in lipid or glucose metabolism could be altered. Consequently, the expression of a selected number of relevant genes involved in metabolism was quantified by RT-qPCR (Table 6).

**TABLE 6:** Impact of embryo origin and maternal diet during gestation on the expression of genes involved in oxidative stress or metabolism in the liver of fetuses 28 dpc.

Recipient diet	Control (C)	Control (C)	High fat (H)	High fat (H)	Kruskal-Wallis test	Dunn’s *post-hoc* comparison test
Embryo origin	dC	dH	dC	dH		
Groups Targeted genes⇓	CC (*n* = 15)	HC (*n* = 16)	CH (*n* = 20)	HH (*n* = 12)	*P*-value	Significant differences between groups
Rabbit AGER	0.0048 [0.0039; 0.0064] a	0.0077 [0.0051; 0.0120] a	0.0002 [0.0001; 0.0007] b	0.0002 [0.0001; 0.0003] b	<0.0001	(CC = HC) > (CH = HH)
Rabbit ALB	562 [520; 638] a	526 [504; 596] a	1139 [889; 1309] b	1113 [950; 1458] b	<0.0001	(CC = HC) < (CH = HH)
Rabbit Adipo	0.93 [0.82; 1.03] a	0.92 [0.62; 1.00] a	0.70 [0.58; 1.02] ab	0.76 [0.66; 0.85]b	0.0017	(CC = HC) > HH
Rabbit CD36	1.34 [1.09; 1.57]	1.32 [1.10; 1.60]	1.14 [0.90; 1.48]	1.17 [0.99; 1.30]	0.4716	ns
Rabbit ELOVL6	0.031 [0.024; 0.039] a	0.029 [0.026; 0.034] a	0.011 [0.008; 0.016] b	0.010 [0.008; 0.011] b	<0.0001	(CC = HC) > (CH = HH)
Rabbit FADS1	0.18 [0.16; 0.21] a	0.18 [0.17; 0.22] a	0.14 [0.07; 0.21] b	0.11 [0.09; 0.12] b	<0.0001	(CC = HC) > (CH = HH)
Rabbit FADS2	0.38 [0.30; 0.46] a	0.39 [0.37; 0.50] a	0.18 [0.147; 0.24] b	0.15 [0.13; 0.19] b	0.0005	(CC = HC) > (CH = HH)
Rabbit LDL-R	0.12 [0.09; 0.15] a	0.12 [0.11; 0.13] a	0.06 [0.04; 0.09] b	0.06 [0.05; 0.07] b	<0.0001	(CC = HC) > (CH = HH)
Rabbit LXR α	0.109 [0.101; 0.124] a	0.113 [0.097; 0.126] a	0.077 [0.068; 0.094] b	0.081 [0.071; 0.098] b	0.0001	(CC = HC) > (CH = HH)
Rabbit PPARα	0.20 [0.18; 0.24] a	0.24 [0.22; 0.25] a	0.10 [0.08; 0.14] b	0.12 [0.10; 0.13] b	<0.0001	(CC = HC) > (CH = HH)
Rabbit IR	0.38 [0.35; 0.44] a	0.40 [0.38; 0.47] a	0.19 [0.15; 0.28] b	0.21 [0.18; 0.23] b	<0.0001	(CC = HC) > (CH = HH)
Rabbit IRS1	0.025 [0.023; 0.026] a	0.025 [0.021; 0.028] a	0.011 [0.007; 0.013] b	0.009 [0.008; 0.011] b	<0.0001	(CC = HC) > (CH = HH)
Rabbit FAS	0.092 [0.073; 0.133] a	0.122 [0.095; 0.144] a	0.018 [0.012; 0.026] b	0.017 [0.013; 0.026] b	<0.0001	(CC = HC) > (CH = HH)
Rabbit GAB2	0.006 [0.005; 0.007] a	0.005 [0.004; 0.007] a	0.002 [0.001; 0.005] b	0.002 [0.001; 0.002] b	<0.0001	(CC = HC) > (CH = HH)
Rabbit PCTP	0.0129 [0.0121; 0.0161] a	0.0165 [0.0129; 0.0216] a	0.0014 [0.0011; 0.0032] b	0.0015 [0.0010; 0.0017] b	<0.0001	(CC = HC) > (CH = HH)
Rabbit SCD5	0.015 [0.013; 0.017] a	0.012 [0.010; 0.016] a	0.004 [0.003; 0.005] b	0.002 [0.002; 0.003] b	<0.0001	(CC = HC) > (CH = HH)
Rabbit SREBP2	0.116 [0.094; 0.122] a	0.102 [0.092; 0.118] a	0.045 [0.030; 0.049] b	0.042 [0.037; 0.049] b	<0.0001	(CC = HC) > (CH = HH)

*Relative gene expression obtained by RT-qPCR (2^–ΔCt^) of genes encoding advanced glycosylation end product-specific receptor (AGER or RAGE), albumin (ALB), adiponectin (Adipo), differentiation cluster glycoprotein 36 (CD36), fatty acid elongase 6 (ELOVL6), delta 5 and 6 desaturases (FADS1 and FADS2), low-density lipoprotein receptor (LDL-R), nuclear receptors Liver X receptor alpha (LXRα), retinoic X receptor alpha (RXRα), insulin receptor IR), insulin receptor substrate 1 (IRS1), fatty acid synthase (FAS), associated binding protein 2 (GAB2), phosphatidylcholine transfer protein (PCTP), stearoyl-CoA desaturase 5 (SCD5) and sterol regulatory element-binding protein 2 (SREBP2) in the liver of fetuses 28 dpc. The values were normalized with DataAssists Software, using two reference genes coding for 4E translation initiation factor 2 (EIF4E2) and ribosomal protein L18 (RPL18). Statistically, the data were analyzed by Kruskal-Wallis test followed by, if applicable (i.e., if significant at p < 0.05), pairwise Dunn’s multiple comparisons test to explore preconception exposure (embryo origin) and in utero exposure (recipient diet during gestation). The data are represented by Median [Q1; Q3] as the median, the 1st and 3rd quartiles. Medians in a row without a common letter differ, P < 0.05. CC, control embryo transferred in control recipient does; CH, control embryo transferred in HD-fed recipient does; HC, embryo from HD-fed donor transferred in control recipient does; HH, embryo from HD-fed donor transferred in HD-fed recipient does.*

The gene expression of albumin (ALB) was increased in the CH and HH groups compared to CC and HC. The gene expression of adipophilin was significantly reduced in the CH and HH groups compared to those raised by C recipients (CC and HC). CD36 gene expression did not differ between groups. All the other genes studied, whether involved in lipid or glucose metabolism, were downregulated in CH and HH (Table 6).

**FIGURE 8:**
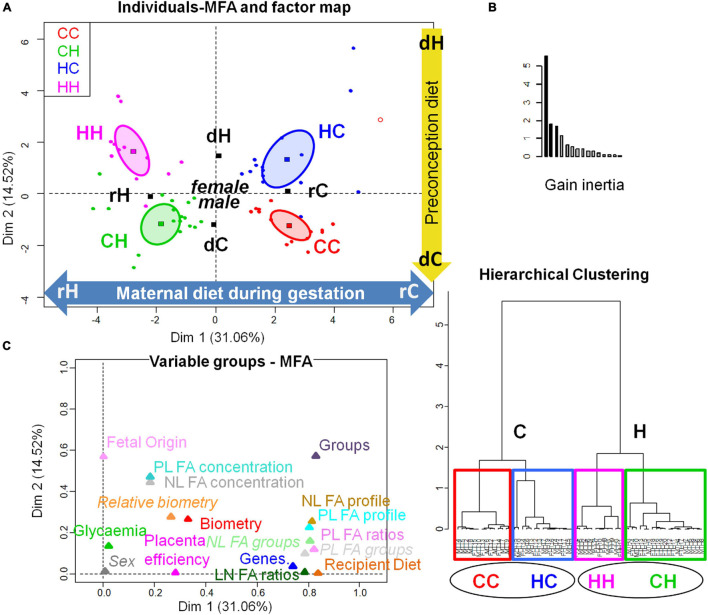
Multifactorial analysis of the combination of feto-placental biometry, fetal liver gene expression, and fetal liver FA signatures and concentrations. **(A)** Individuals-MFA and factor map (with confidence ellipses around group categories). **(B)** Hierarchical clustering (with graph of inertia gain). **(C)** Variable groups-MFA.CC, embryo from control donor transferred in control recipient does; CH, embryo from control donor transferred in HD-fed recipient does; HC: embryo from HD-fed donor transferred in control recipient does; HH, embryo from HD-fed donor transferred in HD-fed recipient does; dC, control embryo donor; dH, H embryo donor; rC, C recipient does; rH, H recipient does. The plot Variable groups-MFA illustrates the correlation between groups and dimensions. On the one hand, recipient diet correlated with dimension 1 (cos^2^ = 0.948, | v.test| = 6.84, positively with rC and negatively with rH) with a positive contribution of several gene expressions (IRS1, SREBP2, SCD5, PCTP, IR, and PPARalpha), NL- and PL- DHA, and long-chain MUFA (C18, C20, and C24, with *p-*values > 1e-07), and a negative contribution of NL- and PL-linoleic and alpha-linolenic acids, ω6 PUFA, PUFA-to-SFA ratio, and ω6-to-ω3 PUFA ratio. On the other hand, fetal origin correlated with dimension 2 (cos^2^ = 0.599, | v.test| = 5.258, positively with dH and negatively with dC) with a positive contribution of NL- and PL-MUFA, NL and PL concentrations, and delta6 and delta9DAI, and a negative contribution of liver and kidney biometry, NL- and PL-SFA and long-chain ω6 PUFA (C22). The Lg coefficients of relationship between the tables allow to measure to what extent the tables are related two by two. The more variables of a first table are related to the variables of the second table, the higher the Lg coefficient, this is represented by their proximity on the graph C.

The MFA combining biometric data sets and fetal liver gene expression with fetal liver (NL and PL) fatty acid concentration and profiles showed that recipient diet played a preponderant role, although a clear separation among the four groups could be observed either on the factor map (Figure 8A) or hierarchical clustering (Figure 8B). These data strengthened the effects observed on liver weight, (CC > HH, with HC and CH in between). The FA profiles or gene expression in HC were not significantly different from CC, when we studied these different datasets separately, however, in this MFA, this HC group appeared completely dissociated from CC, demonstrating that the fetal repercussions of the preconception environment also deserved to be studied because it affects, beyond the placenta, the fetus health as well. The plot Variable groups-MFA illustrates the correlation between groups and dimensions (Figure 8C). On the one hand, recipient diet correlated with dimension 1, positively with rC correlated with the positive contribution of several gene expressions (IRS1, SREBP2, SCD5, PCTP, IR, and PPAR alpha), NL- and PL- DHA, and long chain MUFA (C18, C20, and C24), and negatively with rH correlated with the negative contribution of NL- and PL-linoleic and alpha-linolenic acids, ω6 PUFA, PUFA-to-SFA ratio, and ω6-to-ω3 PUFA ratio. On the other hand, embryo origin correlated with dimension 2, positively with dH correlated with the positive contribution of NL- and PL-MUFA, NL and PL concentrations, and delta6 and delta9DAI, and negatively with dC correlated with the negative contribution of liver and kidney biometry, NL- and PL-SFA, and long chain ω6 PUFA (C22).

## Discussion

### Context

The objective of this study was to discriminate the effects of maternal lipid over-nutrition between effects due to a pre-conceptional period of exposure and those due to gestational exposure only. The rabbit model was used because of its metabolic (sensitivity to lipids) and anatomical (hemodichorial placenta) particularities similar to those of humans (Fischer et al., 2012). This study is the follow-up of a previous one, where dams fed with a high-fat diet (HD) started shortly after weaning were shown to have precocious puberty and increased follicular atresia (Cordier et al., 2013). In addition, embryos produced by these dams after mating with control males were characterized by deregulation of gene expression at the onset of embryonic genome activation (8–16 cells) (Picone et al., 2011) and in the blastocyst stage were observed near to term (Tarrade et al., 2013). Offspring postnatal phenotype was characterized by predisposition to develop metabolic syndrome, such as excess adiposity and hypertension (Picone et al., 2011; Dupont et al., 2014). However, since maternal exposure to high-fat diet extended from puberty to the end of gestation, it was not possible to dissociate the effect of each period of exposure on the feto-placental phenotype, i.e., an early effect, affecting the gametogenesis of rabbits from a pure gestational effect of this exposure. In this study, the fetoplacental effects of maternal exposure to a high-fat diet were investigated for each window of vulnerability and a combination of the two. Thereby, data from C embryos or H embryos transferred in C recipient females are designated by CC and HC, respectively, and those from C embryos or H embryos transferred in H recipient females are designated by CH and HH, respectively.

As a programming agent of offspring phenotype, the placental phenotype was investigated at the end of gestation. Both placenta and decidua were targeted to explore placental adaptations in response to the maternal environment and flexibility of the maternal decidua in response to embryo origin. The CC and HH groups were used the negative and positive controls, respectively, to confirm the fetal hypotrophy with significantly reduced placental efficiency previously observed in fetuses from high-fat diet-fed dams (Tarrade et al., 2013), and to exclude any significant effect of reproductive biotechnologies related to embryo transfers. Without any post-natal end point, in this study, possible alterations of offspring phenotype were investigated in the fetal stage, on fetal plasma and liver. Maternal high-fat diet during the preconception period and gestation periods, i.e., the HH group led to growth-retarded fetuses in rabbit. In the literature, the effects of a high-fat maternal diet on birth weight are variable, sometimes leading to no effect or higher birth weight, for example in rats, depending on the period of exposure (during the entire gestation or the last third of the gestation) and qualitative composition of dietary lipids, especially when the diet is saturated (Cerf et al., 2015; Seet et al., 2015).

While epigenetic marks are currently described as major candidates for predicting the long-term health of individuals (Tarrade et al., 2015), the importance of other biochemical parameters might have been overlooked. Among these, fatty acid (FA) profiling makes it possible to obtain a fine phenotype at the end of gestation to highlight the differences between groups according to the windows of exposure to excessive lipid intake in the mother, i.e., before and/or during pregnancy. FAs are described as one of the most essential substances in intrauterine human growth, through their involvement in a number of energetic and metabolic processes, such as the growth of cell membranes (Bobinski and Mikulska, 2015; Shrestha et al., 2020b). Essential FAs (EFAs), namely, linoleic acid (LA, ω6 series) and alpha-linolenic (ALA, ω3 series), and their derivatives, namely, arachidonic acid (AA, C20:4ω6) and docosahexaenoic acid (DHA, C22:6ω3), are among the most important FAs during the intrauterine growth period.

Linoleic acid is highly required during gestation as a precursor of arachidonic acid (and metabolites, i.e., prostaglandins) strongly involved in the development of embryos and placentation, with a positive association with fetal growth when combined with ω3 PUFA (Badart-Smook et al., 1997). However, excessive consumption of ω6 PUFA, i.e., excessive LA, leads to a negative association to fetal growth PUFA (Badart-Smook et al., 1997), by altering cellular metabolism through inflammation or modulation of mitochondrial function (Cetin et al., 2009). Fetal LA is obtained from the maternal diet, and FAs are transported to the fetus through placental FA transporters (FATPs) and binding proteins (FABPs). Moreover, it was recently reported that consumption of ω6 PUFA, i.e., LA, has increased gradually worldwide (Choque et al., 2014), with a potential harmful effect on human health, especially before or during pregnancy, leading to detrimental effects on fetal development that could influence offspring health at adulthood (Shrestha et al., 2020b). LA is a direct precursor of bioactive oxidized linoleic acid metabolites described to promote a strong pro-inflammatory response in rats (Choque et al., 2014). LA is also a precursor of arachidonic acid, which is the substrate of several enzymes leading to the production of pro-inflammatory eicosanoids (prostaglandin E2, thromboxane, and 4 series-leukotrienes). Therefore, excessive consumption of LA during pregnancy may, thus, improve its conversion to AA and unbalance eicosanoid synthesis (Elmes et al., 2004; Choque et al., 2014).

### Placental Phenotype

**FIGURE 9:**
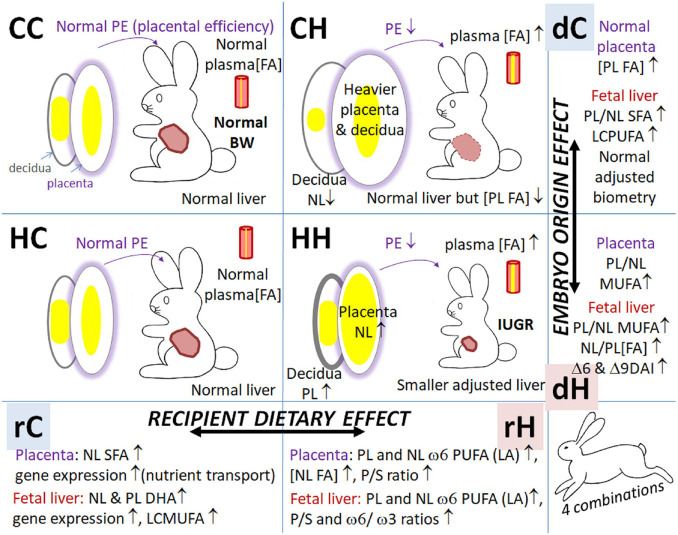
Summary of feto-placental effects of maternal dietary exposure during either preconception or gestational window. CC, embryo from control donor transferred in control recipient does; CH, embryo from control donor transferred in HD-fed recipient does; HC, embryo from HD-fed donor transferred in control recipient does; HH, embryo from HD-fed donor transferred in HD-fed recipient does; dC, control donor; dH, H embryo donor; rC, control recipient does; rH, H recipient does. BW, body weight; FA, fatty acids; IUGR, intrauterine growth retardation; LCMUFA, long-chain monounsaturated fatty acids; NL, neutral lipids (i.e., intracellular lipid storage); PE, placental efficiency; PL, phospholipids (i.e., membranes); P/S, polyunsaturated-to-saturated fatty acid ratio; PUFA, polyunsaturated fatty acids; SFA, saturated fatty acids.

Maternal dietary-induced overweight and adiposity are usually characterized by maternal dyslipidemia. In fact, our previous studies have demonstrated abnormalities in lipid storage in term placentas (Tarrade et al., 2013). However, there is little information on the effect of pre-gestational overweight with excessive fat on placental lipid metabolism. The transfer of both C and H embryos in H females led to a reduction in placental efficiency. Nevertheless, the feto-placental phenotype also differed according to the origin of the embryo, as summarized in Figure 9. Compared to controls (CC), placentas were heavier in the CH group, without significant impairment of fetal or organ weight. On the other hand, compared to the CC group, placentas, and fetuses and their organs (liver and kidneys) were lighter in the HH group. The significant decrease in kidney weight, both in raw and relative to body weight values, could explain the hypertension previously observed in adult male offspring born to HH dams (Picone et al., 2011; Dupont et al., 2014).

In this study, LA-enriched HD significantly increased the fetal liver NL and plasma AA contents in the CH and HH groups, as already shown in ewes where LA supplementation in late pregnancy was shown to enhance placental PG production by increasing placental AA supply (Elmes et al., 2004). Moreover, exposure to HD throughout gestation led to the downexpression of genes involved in nutrient transport, namely glucose transporters (SLC2A1 and 3) or amino-acid transporters (SLC38A1, 2, and 3) as well as FATP4, whose role is to enhance FA internalization for transplacental transfers (Bobinski and Mikulska, 2015). Similar observations were reported on rats where a high maternal LA diet altered the placental FA composition (increased ω6 PUFA and decreased DHA), and increased the placental gene expression of inflammatory proteins and nutrient transporters, with a downregulation of fatty acid transport protein 4 (Fatp4) and glucose transporter 1 (Slc2a1) (Shrestha et al., 2020a).

Most studies show downregulation of key placental transporters for amino acids, lipids, and ions in human IUGR (Duttaroy and Basak, 2020), as seen here in the HH hypotrophic rabbit fetuses. These observations underlie the placental nutrient sensor model and fetal demand model hypotheses stipulating that the placenta integrates maternal and fetal nutritional cues with information from intrinsic nutrient sensors (Gaccioli et al., 2013); thus the placenta would play a critical role in modulating maternal-fetal resource allocation, thereby affecting fetal growth and the long-term health of the offspring. This hypothesis is corroborated here by the response of decidua membrane to embryo origin (increased FA concentration in deciduous PL of HH and, more slightly, in HC). Here, maternal HD during gestation induced increased FA concentration inside the labyrinthine area (in NL lipid fraction). In H recipients, the proportion of ω6 PUFA, such as LA and ALA (ω3 PUFA), in placental lipids (both PL and NL lipid fractions), along with increased PUFA-to-SFA ratio, was greater than in C recipients.

Pre-conceptional exposure to high fat-diet had a major effect on fetal biometry and organogenesis (lighter liver and kidneys) in HH. It also altered FA concentrations of placenta phospholipids, involved in membrane synthesis, as well as increased the MUFA contents in the NL lipid fraction, as revealed by placental MFA. This phenotype, i.e., the accumulation of lipids, was already observed at the embryo level (Picone et al., 2011) and in trophoblastic cells (Tarrade et al., 2013). Thus, an H embryo transferred to a C recipient (HC) evolved favorably in terms of biometrical development, which seemed similar to controls (CC) at term, with, however, a small but significant effect on placental profiles of fatty acids. This MFA analysis shows notably that the maternal diet during gestation plays a dominant role on the feto-placental phenotype (biometry and lipid signature) but that pre-conception maternal environment undeniably remains very important in term of programming.

### Fetal Phenotype

In tissues, LA and ALA, which make up for the major part of dietary EFA, are converted into FA of longer and more unsaturated chain by alternate desaturation (delta 6, delta 5)-elongation reactions. The liver is one of the most active organs, and its role is critical in providing less active tissues, particularly the brain, with long-chain PUFA secreted and transported in VLDL (very low density lipoprotein) (Bezard et al., 1994). In this study, maternal HD had different impacts on fetal liver phenotype depending on time windows of exposure, as revealed by the fetal liver MFA.

Excessive ω6 PUFA consumption, together with high ω6/ω3 PUFA ratio (i.e., low intake of ω3 PUFA), is highly associated with the pathogenesis of many modern diet-related chronic diseases (Mariamenatu and Abdu, 2021) and could lead to a pro-inflammatory environment. In this study, the linoleic acid-rich HD significantly increased the AA content of fetal plasma and fetal liver NL in the CH and HH groups, as already shown in ewes in which LA supplementation in late pregnancy enhanced placental PG production by increasing the supply of AA (Elmes et al., 2004). In comparison to C recipients, in the H recipient groups (CH and HH), fetal liver lipids (both PL and NL lipid fractions), as for placenta, had a greater proportion of ω6 PUFA, such as LA, and of ALA with an increase in the PUFA-to-SFA and ω6-to-ω3 ratios, whereas DHA and long-chain MUFAs (C18:1, C20:1, and C24:1) were reduced in liver membranes (PL fraction). In rats, in the context of high-fat diet (HFD), high consumption of LA during pregnancy was reported to affect liver FA profiles of offspring in a similar manner, with increased ω6 PUFA and decreased MUFA (Marchix et al., 2020a). Surprisingly, however, and as previously observed in rabbits (Tarrade et al., 2013) and rats (Shrestha et al., 2021) fed with a high-fat diet rich in LA, FA concentrations in liver neutral lipids were similar among groups.

In parallel, the expression of several genes, among which IRS1, IR, PCTP, SCD5, and PPARα, as well as those involved in lipid metabolism was downregulated in CH and HH in this study. This is in agreement with data reported in rats, where a maternal high-LA diet was shown to lead to reduced hepatic mRNA expression of several genes involved in lipogenic pathway, srebp-1c (*de novo* lipogenesis), Scd-1 (conversion of SFA to MUFA), Fasn (FA synthase), and Elovl6 (FA elongase 6), and to affect the desaturation index (Δ5 desaturase) (Marchix et al., 2020b). Thus, in rats, maternal HF diet during pregnancy was shown to alter the liver C16 desaturase pathway (Seet et al., 2015), as in this study with an increased Δ9 C16 desaturase index. The strong decrease in the hepatic expression of PCTP could be the cause of a possible degradation of glucose homeostasis in fetuses of H recipients (CH and HH), since the increase in circulating PLTP (phospholipid transfer protein) is described as an inter-organ (brown adipose tissue and liver) mediator to improve glucose tolerance and insulin sensitivity (Sponton et al., 2020). Furthermore, in rats, dietary-induced maternal obesity has been shown to program hepatic metabolic anomalies that lead to the development of fatty liver in the offspring through alterations involving PPARα-activated mechanisms (Heinecke et al., 2020).

The expression of genes involved in lipid metabolism, such as FAS, was repressed in the liver of fetuses carried by the H recipients (i.e., CH and HH groups); however, the overexpression of albumin mRNA was also observed in these groups. Albumin reversibly binds fatty acids with high affinity (Naval et al., 1992) and might be involved in the high materno-fetal FA flux across the placenta. In rats, PUFA in fetal plasma and liver are significantly correlated to PUFA in maternal plasma, which is also closely related to the fatty acid composition in the maternal diet (Amusquivar and Herrera, 2003). In addition, rat fetuses exposed throughout gestation to a maternal high-fat diet were shown to have plasma FA profiles rich in ω6 PUFA (Cerf et al., 2015). In the present study, similar effects were observed in fetal plasma from CH and HC groups, with increased LA and DGLA contents, as well as in fetal liver, besides increased LA, eicosatrienoic acid (C20:2ω6) and AA whereas DPA decreased compared to controls.

Again, exposure to HD during the pre-conception period had a major effect on fetal biometry and organogenesis, with repercussions on the fatty acid concentration of fetal liver PL and NL, as reported in a previous study (Higham et al., 1984); it increased the content of MUFA in both PL and NL lipid fractions (in HC vs. CC and in HH vs. CH), and decreased the content of SFA and long-chain (C22) ω6 PUFAs. Thus, an H embryo transferred to a C recipient (HC) evolved favorably in terms of biometrical development, which seems similar to controls (CC) at term, with, however, a small but significant effect on fetal liver fatty acid concentration and biometry data. This MFA analysis also shows that the maternal diet during gestation played a dominant role in fetal liver phenotype (gene expression and lipid signatures).

## Conclusion and Take-Home Message

Studying biochemical and molecular processes that underlie the DOHaD concept is essential to be able to adapt nutritional strategies in the context of a changing dietary environment. Understanding the impact of maternal nutrition, either during pre-conception or gestation, on offspring obesity and health later in life is currently one of the most important scientific challenges. This study supports the results that were focused either on pre-conception or the gestational period. Our results show that the effects are not as clear between the two exposure periods (Figure 9). These two periods indeed present distinct effects, sometimes subtle, that could nevertheless have repercussions on the health of the offspring in adulthood. If HC cannot be said to be similar to CC, the differences that persist between these two groups seem reasonably negligible. On the other hand, CH and HH present rather negative phenotypic profiles. This study highlights the benefit of starting nutritional intervention during the pre-conception period in a context of maternal high-fat-diet-induced overweight to limit fetal and placental outcomes, which are major in the case of over-nutrition during gestation. However, the main limitation of this study is that we were unable to study the sex-specific responses of the feto-placental unit, because the small sample size for the females is too small. In conclusion, our results suggest that, in the context of dietary-induced obesity, interventions, at least during gestation, may be favorable in partially correcting the effects of early exposure of the mother to a high-fat diet on the health of the offspring and, mostly, to preserve the offspring from an excessively rich nutritional intrauterine environment during their critical phase of development.

## Data Availability Statement

The original contributions presented in the study are included in the article/Supplementary Material, further inquiries can be directed to the corresponding author/s.

## Ethics Statement

The animal study was reviewed and approved under N°11/037 by the local ethical committee for animal experimentation COMETHEA (“Comité d’Ethique en Expérimentation Animale du Centre INRAE de Jouy en Josas et AgroParisTech”), referenced as N°C2EA-45 in the French National registry CNREEA (“Comité National de Réflexion Ethique sur l’Expérimentation Animale”).

## Author Contributions

DR-R, AC-T, and PC-P: conceptualization, writing—review, and editing. DR-R, AC-T, ND, MD, and GM: *in vivo* investigations. DR-R, M-CA, MD, and AC-T: sample collection and biometry. DR-R, AC-T, AP, CR, and JB: molecular biology. DR-R: lipid analyses, data curation, formal analyses, and writing—original draft. All authors discussed and granted the results, read, improved, and approved the final version of the manuscript.

## Conflict of Interest

The authors declare that the research was conducted in the absence of any commercial or financial relationships that could be construed as a potential conflict of interest.

## Publisher’s Note

All claims expressed in this article are solely those of the authors and do not necessarily represent those of their affiliated organizations, or those of the publisher, the editors and the reviewers. Any product that may be evaluated in this article, or claim that may be made by its manufacturer, is not guaranteed or endorsed by the publisher.

## Footnotes

1
www.r-project.org/
