# Supplementary material for: Importance of Windows of Exposure to Maternal High-Fat Diet and Feto-Placental Effects: Discrimination Between Pre-conception and Gestational Periods in a Rabbit Model
Source: Front Physiol. 2021 Nov 25;12:784268. doi: 10.3389/fphys.2021.784268 (PMC8656279; doi:10.3389/fphys.2021.784268)
Supplement: Supplementary file 1 [file Data_Sheet_1.PDF]

Supplementary Material

Supplementary Table 1: Fatty acid composition of diets, expressed as qualitative (% of total fatty acids) and quantitative (mg/ g of diet) fatty acid profiles.

Diets	Control diet		High fat diet	
Fatty acids	%	mg/g of diet	%	mg/g of diet
C16:0	15.9	3.09	12. 9	7.67
C18:0	7.4	1.44	5.2	3.08
C18:1ω9	12.6	2.45	21.1	12.56
C18:2 ω6 (LA)	47.3	9.22	49.7	29.56
C18:3 ω6	5.0	0.98	2.3	1.38
C18:3 ω3	10.3	2.01	6.6	3.94
SFA	23.2	4.53	18.1	10.76
MUFA	14.1	2.75	23.3	13.89
ω6 PUFA	52.3	10.20	52.0	30.94
ω3 PUFA	10.3	2.01	6.6	3.94
ω6/ω3 PUFA ratio	5		8	

Supplementary Figure 1: Fatty acid composition of control (blue) and high fat (red) diets, expressed as qualitative (% of total fatty acids, on the left side) and quantitative (mg/ g of diet, on the right side) fatty acid profiles.

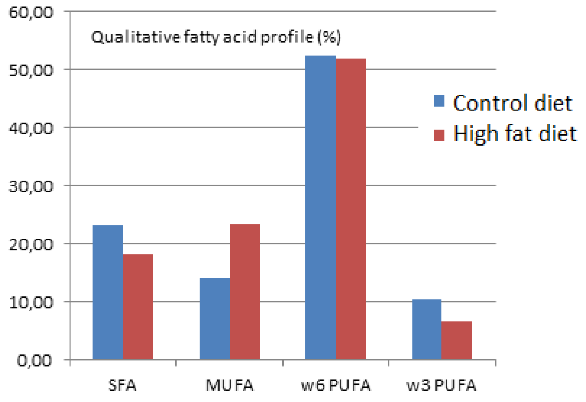

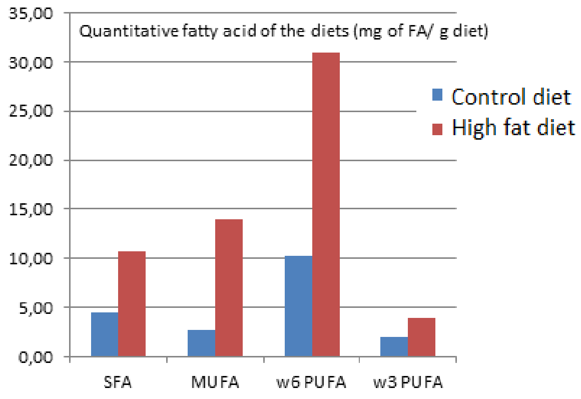

Supplementary Table 2: Primer sequences

Genes	Accession number	Oligo name	Sequence
Rabbit EIF4E2	ENSOCUG00000004534	R-FW-EIF4E2<br>R-RV-EIF4E2	TGGCAAGTGGATTATTCGGC<br>CAGAGACCACAGCCCCACAG
Rabbit RPL18	DQ403030	R-FW-RPL18<br>R-RV-RPL18	CAACTCCACGTTCAACCAGGT<br>GGTCTTGTTCTCCCGCCC
Rabbit Adipophilin	CU465044	R-FW-adipophilin<br>R-RV-adipophilin	GGGCCAGAGTTTCTGTAGCCA<br>CCCAAGACTGTGTTAATGCTGC
Rabbit CD36	AF412572	R-FW-CD36<br>R-RV-CD36	TGCTGCAGTTCTTTTCCTCTGA<br>GGAGATGCAAAAGCCTTGGC
Rabbit IR	AY339877.1	R-FW-IR<br>R-RV-IR	ACCGACTACCTGCTGCTGTT<br>TGACCAGCGCATAGTTGAAG
Rabbit LDL-R	XM_002723277	R-FW-LDR-R<br>R-RV-LDR-R	GTGCAACTCCGCCAGGGA<br>GCCGATTCTGAGGTCGAAGC
Rabbit LXR-α	AB536719	R-FW-LXR-a<br>R-RV-LXR-a	AGGATTTCAGCTACAACCGGG<br>GCGAACTCGGCATCATTGAG
Rabbit PPAR-α	XM_002723354.3	R-FW-PPAR-a<br>R-RV-PPAR-a	TTGTGGCTGCTATCATCTGC<br>GAGTTTGGGGAAGAGGAAGG
Rabbit ALB	Gene ID 100009195	R-FW-ALB<br>R-RV-ALB	TCGACAGACGACCATGCTTTAG<br>TCCTCTCCGTTTCTGGAAGAGT
Rabbit IRS1	Gene ID 100339981	R-FW-IRS1<br>R-RV-IRS1	ATCCCCACCATCATGTCCTA<br>ACATACTCCCCTGGGCTCTT
SREBP2	AF278693	R-FW-SREBP2<br>R-RV-SREBP2	GCTCGAGCCTCCCAAAGAAG<br>GGCATCTGTCCCCATGACC
Rabbit AGER (RAGE)	Ref. Vignozzi L. et al 2012	R-FW-AGER<br>R-RV-AGER	GCAGTCAGAGCTGATGGTGA<br>GCTACTGCTCCACCTTCTGG
Rabbit FADS1	ENSOCUG00000014364	R-FW-FADS1<br>R-RV-FADS1	TTTGGGACGTCCTTAGTGCC<br>GTGCATGTGGTTCCACCAAC
Rabbit FADS2	ENSOCUG00000003499	R-FW-FADS2<br>R-RV-FADS2	ACCCTACCGAGACTGGTTCA<br>GCGATCTTGTGCAGGTTGTG
Rabbit ELOVL6	ENSOCUG00000013069	R-FW-ELOVL6<br>R-RV-ELOVL6	GCTGATCTTCCTGCACTGGT<br>CTTCCGGGAGACTCGGAAAC
Rabbit GAB2	ENSOCUG00000017463	R-FW-GAB2<br>R-RV-GAB2	TGGACAACATGGACCTTCCG<br>TCGAGGTGTTTCTGCCTGAC
Rabbit SCD5	XM_008267676.2	R-FW-SCD5<br>R-RV-SCD5	CAAGCATCCAGATGTCATCGA<br>CGCTGATCTTATAGTACTTTCTCTGGAA
Rabbit PCTP	ENSOCUG00000008956	R-FW-PCTP<br>R-RV-PCTP	CCCTTTCCCCATGTCCAACA<br>CTTTGCTTCCCATCTTGCCG
Rabbit β actin	NM_001101683	R-FW-BACT<br>R-RV-BACT	CGAGACCACCTTCAACTCGATC<br>CTTCTGCATGCGGTCGG
Rabbit ABC-A1	XM_002708133	R-FW-ABC-A1<br>R-RV-ABC-A1	GGTGATGAGCCGGTCAATG<br>CCATGATCCGCATGGTCTC
Rabbit ABC-G1	ENSOCUT00000027993	R-FW-ABC-G1<br>R-RV-ABC-G1	TCATCCTGTCCATCTACGGCC<br>TGCAACTTGGCGTTCTCCAC
Rabbit FAS	ENSOCUT00000022126	R-FW-FAS<br>R-RV- FAS	ACTACAACCTCTCGCAGGTGTG<br>AGGGAGCTGTGCATGATGC
Rabbit FATP-4	XM_002722970.1	R-FW-FATP-4<br>R-RV-FATP-4	AGGAGCTGCCCCTGTATGC<br>CACGACAGCTGGGTCAAAGC
HMG-coA reductase	XM_002723033	R-FW-HMG-coA<br>R-RV-HMG-coA	GACTCCCCACACAGAGCTGC<br>ATTCTTCATTAGGCCGAGGCT
RXR alpha	AF136242	R-FW-RXRa<br>R-RV-RXRa	CAAGGAGAGGAACGAGAACG<br>CACGTAGGTCTCGGTCTTGG
Rabbit PPAR-γ	NM_001082148	R-FW-PPAR-g<br>R-RV-PPAR-g	TGAACGACCAGGTGACTCTGC<br>TCCCTCGTCATGAAGCCTTG
Rabbit SLC2A1	NM_001105687	F-FW-SLC2A1<br>F-RV-SLC2A1	ACCACGCTGTGGTCCCTCT<br>GCAGGTTCATCATCAGCATTGA
Rabbit SLC2A3	XM_002712761	F-FW-SLC2A3<br>F-RV-SLC2A3	AGAAGGAAGAGGACGAGGCC<br>GTGACTTGCTTCTCCTGGGC
Rabbit SLC38A1	ENSOCUT00000005167	R-FW-SLC38A1<br>R-RV-SLC38A1	GCGTGCACACCAAGATACGT<br>ACCGATCCTTAAGCTCGCTG
Rabbit SLC38A2	ENSOCUT00000009942	R-FW-SLC38A2<br>R-RV-SLC38A2	ATTGTCCGACTGGCTGTGCT<br>CTGTGACGCCACCAACTGAA
Rabbit SLC38A4	ENSOCUT00000026469	R-FW-SLC38A4<br>R-RV-SLC38A4	GGATGCAGACGGTGTCCAAC<br>CTGTAGGCGTGCAGCAGTTC

ABC-A1 and ABC-G1: ATP-binding cassette A1 and subfamily G member 1 for the transport of cholesterol; Adipo: adipophilin; AGER (RAGE): advanced glycosylation end product-specific receptor; ALB: albumin; CD36: differentiation cluster glycoprotein 36 known as fatty acid translocase; ELOVL6: fatty acid elongase 6; GAB2: associated binding protein 2; FADS1 and FADS2: delta 5 and 6 desaturases; FAS: fatty acid synthase; FATP4: fatty acid transport protein 4;HMG CoA: 3-hydroxy-3-méthylglutaryl-coenzyme A reductase, LDL-R: low-density lipoprotein receptor; LXRα: nuclear receptors Liver X receptors; IR: insulin receptor; IRS1: insulin receptor substrate 1; PCTP: phosphatidylcholine transfer protein; PPARγ: peroxisome proliferator-activated receptor gamma; RXRα: retinoic X receptor alpha; SCD5: stearoyl-CoA desaturase 5SLC2A1: solute carrier family 2 (facilitated glucose transporter) member 1 known as GLUT-1; SLC2A3: GLUT-3; SLC38A1, SLC38A2 and SLC38A4: sodium-coupled neutral amino acid transporter 1, 2 and 4; SREBP2: sterol regulatory element-binding protein 2; and 2 housekeeping reference genes, with EIF4E2: 4E translation initiation factor 2 and RPL18: ribosomal protein L18.
